# Targeting ATR and PI3Kα Pathways Promotes Ferroptosis in *PIK3CA*-Wildtype Platinum-Resistant Endometrial Cancer

**DOI:** 10.3390/cancers18071064

**Published:** 2026-03-25

**Authors:** Chi-Ting Shih, Kristen R. Ibanez, Jung-Min Lee, Tzu-Ting Huang

**Affiliations:** 1 Women’s Malignancies Branch, Center for Cancer Research (CCR), National Cancer Institute (NCI), National Institutes of Health (NIH), Bethesda, MD 20892, USA; chi-ting.shih@nih.gov (C.-T.S.); kristen.ibanez2@mountsinai.org (K.R.I.); jamie9298@gmail.com (J.-M.L.); 2 GlaxoSmithKline (GSK), Collegeville, PA 19426, USA

**Keywords:** endometrial cancer, ferroptosis, chemoresistance, ATR inhibitor, PI3K inhibitor

## Abstract

**Background/Objectives:** Platinum resistance in endometrial cancer (EC) remains a significant therapeutic challenge, as tumors frequently bypass apoptotic cell death. Ferroptosis, an iron-dependent form of regulated cell death driven by lipid peroxidation, offers an alternative mechanism to target apoptosis-resistant cancers. This study evaluated whether combined inhibition of ATR and PI3Kα could induce cell death in platinum-resistant EC through apoptotic or ferroptotic pathways. **Methods:** A panel of EC cell lines, including patient-derived models with varying *PIK3CA* mutation status and platinum sensitivity, was treated with camonsertib (ATR inhibitor) and inavolisib (PI3Kα inhibitor). Cell death mechanisms were assessed through DNA damage indicators (γH2AX, comet assay, DNA fiber analysis), apoptosis markers (Annexin V, cleaved PARP, cleaved caspase 3), and ferroptosis markers (FerroOrange, xCT expression, redox homeostasis). **Results:** While monotherapies showed limited activity, dual ATR and PI3Kα inhibition produced additive/synergistic cytotoxicity across all EC cell lines, independent of platinum sensitivity or microsatellite stability status. Mechanistically, the treatment induced genotype-specific cell death: *PIK3CA*-mutant cells underwent apoptosis driven by catastrophic DNA damage accumulation, whereas *PIK3CA*-wildtype cells exhibited predominantly ferroptosis characterized by xCT downregulation and redox disruption. **Conclusions:** Our findings establish dual ATR and PI3Kα inhibition as a genotype-informed therapeutic strategy for platinum-resistant EC. *PIK3CA* mutation status may influence the mode of cell death, supporting its use as a predictive biomarker for patient stratification in future clinical applications.

## Simple Summary

Platinum resistance limits treatment options in endometrial cancer, as tumor cells evade conventional apoptotic cell death. We demonstrate that dual inhibition of ATR and PI3Kα exerts potent cytotoxicity across all tested endometrial cancer cell lines, independent of *PIK3CA* mutation status. Furthermore, the combination treatment triggers distinct, genotype-specific cell death mechanisms: apoptosis in *PIK3CA*-mutant cells through DNA damage accumulation, and ferroptosis-dominant cell death in *PIK3CA*-wildtype cells via the disruption of antioxidant defenses. Collectively, these findings suggest that combined ATR and PI3Kα inhibition represents a promising therapeutic strategy for platinum-resistant endometrial cancer and establish *PIK3CA* mutation status as a predictive biomarker for treatment selection.

## 1. Introduction

Endometrial cancer (EC) is the sixth most common malignancy among women in the United States, and its incidence and mortality continue to rise worldwide [1,2]. Although most patients are diagnosed at an early stage, the five-year overall survival rate drops to approximately 15% in advanced disease [3]. Among EC subtypes, the serous-like copy number high (CNH) subtype accounts for a quarter of cases and is associated with poorer prognosis and higher rates of platinum resistance [4]. Platinum-based chemotherapy remains the standard of care for late-stage or metastatic EC [5], but responses are often transient and resistance inevitably develops. A key mechanism underlying platinum resistance is the evasion of apoptosis [6,7]. Therefore, new therapeutic approaches that bypass apoptotic resistance mechanisms are urgently needed.

Ferroptosis, an iron-dependent form of regulated cell death characterized by lipid peroxidation [8], has emerged as a promising alternative to overcome apoptosis evasion in therapy-resistant cancers [8,9]. In EC, ferroptosis sensitivity is governed by key regulators including the glutamate/cystine antiporter solute carrier family 7 member 11 (SLC7A11, also called xCT), glutathione peroxidase 4 (GPX4), and enzymes involved in glutathione (GSH) synthesis, which are aberrantly expressed in malignant endometrium [10,11]. Mechanistically, xCT imports cystine to support GSH synthesis, and GSH, together with GPX4, detoxifies lipid peroxides to prevent ferroptosis [12]. Preclinical studies have demonstrated that compounds such as juglone [13] and dihydroisotanshinone I [14] can induce ferroptosis in EC cells, validating ferroptosis as a therapeutically exploitable vulnerability. However, despite these promising preclinical findings, most ferroptosis inducers remain in early-stage development, and no ferroptosis-targeted therapy has yet entered clinical investigation. This gap highlights the need to identify clinically actionable, druggable regulators of ferroptosis in EC.

The phosphatidylinositol 3-kinase (PI3K)/protein kinase B (AKT) pathway represents one such targetable regulator. Frequently dysregulated in up to 50% of EC cases through *PIK3CA* mutations [15], this pathway plays a dual role in promoting tumor survival and suppressing ferroptosis. Mechanistically, constitutive PI3K/AKT activation enhances antioxidant defense by upregulating xCT and GPX4, thereby maintaining redox homeostasis and preventing lipid peroxidation [16]. Pharmacologic inhibition of PI3K may therefore restore ferroptosis sensitivity in EC. A complementary therapeutic strategy involves targeting the ataxia telangiectasia and Rad3-related kinase (ATR). CNH ECs, which frequently lack functional G1/S checkpoints due to *TP53* mutation, are particularly dependent on ATR-mediated G2/M signaling to manage replication stress and maintain genomic stability [17]. Importantly, emerging evidence reveals that ferroptosis and DNA damage response (DDR) pathways are mechanistically interconnected: ATR regulates ferroptosis resistance through stabilization of ubiquitin-specific peptidase 20 (USP20) [18], and conversely, ferroptosis inducers can trigger DDR activation [19,20,21]. This molecular crosstalk suggests that simultaneous inhibition of PI3K and ATR may synergistically exploit both metabolic and replication vulnerabilities in EC.

In this study, we investigated the therapeutic potential of combining the ATR inhibitor (ATRi) camonsertib with the PI3Kα inhibitor (PI3Kαi) inavolisib in a panel of EC cell lines and patient-derived cell (PDC) models with different molecular backgrounds and platinum sensitivities. We demonstrate that dual inhibition produces additive/synergistic cytotoxicity across all EC models, independent of platinum sensitivity or microsatellite stability status, but through distinct, *PIK3CA* genotype-specific mechanisms: apoptosis in *PIK3CA*-mutant (*PIK3CA*m) cells driven by catastrophic DNA damage accumulation, and predominantly ferroptosis in *PIK3CA*-wildtype (*PIK3CA*wt) cells mediated by antioxidant pathway suppression. These findings uncover a previously unrecognized divergence in cell death pathways associated with *PIK3CA* mutation status and provide a mechanistic rationale for biomarker-guided application of ATR and PI3K co-inhibition in platinum-resistant EC.

## 2. Materials and Methods

### 2.1. Cell Lines

Microsatellite instability-high (MSI-h) AN3CA (#HTB-111) and HEC-1-A (#HTB-112) were obtained from the American Type Culture Collection (ATCC, Rockville, MD, USA), while MFE-296 (#98031101) and Ishikawa (#99040201) were from MilliporeSigma (Rockville, MD, USA). CNH-like EC cell lines, including KLE (#CRL-1622, ATCC), MFE-280 (#98050131, MilliporeSigma), and ARK1 and ARK2 (provided by Dr. Alessandro D. Santin, Yale University School of Medicine, New Haven, CT, USA), were used in this study. All cell lines were grown in RPMI 1640 medium with (+) L-glutamine supplemented with 10% fetal bovine serum, 0.01 mg/mL insulin, and 1% penicillin/streptomycin.

Two serous EC PDCs, 354T (922993-354-T-J3-PDC) and 144R (598228-144-R-J1-PDC), were obtained from the NCI Patient-Derived Models Repository (Frederick, MD, USA). PDC lines were cultured in advanced DMEM/F12 supplemented with 5% fetal bovine serum, 1 μg/mL hydrocortisone, 1 μg/mL hEGF, 2.4 μg/mL Adenine, 200 μM L-glutamine, 10 μM Y-27632 dihydrochloride, and 1% penicillin/streptomycin.

### 2.2. Drug Preparation

ATRi camonsertib, PI3Kαi inavolisib, and ferroptosis inducer (imidazole ketone erastin, IKE) were obtained from the NCI Development Therapeutics Program (Frederick, MD, USA). Bortezomib was obtained from Selleck Chemicals (#S1013, Houston, TX, USA). The caspase inhibitor z-VAD-FMK and ferroptosis inhibitor ferrostatin-1 were obtained from MedChemExpress (Monmouth Junction, NJ, USA). All drugs were prepared as separate 10 mM stocks in dimethyl sulfoxide (DMSO; #S-002-M, MilliporeSigma) and stored in aliquots at −80 °C until use.

### 2.3. Cell Viability Assay

A total of 2000–3000 cells/well were seeded in 96-well plates and treated with each drug alone, combination treatments or 0.01% DMSO as a control after seeding. For apoptosis rescue experiments, cells were pretreated with or without z-VAD-FMK, followed by a 72 h drug treatment. After being treated with drugs for 72 h, the cell viability was assessed by the XTT assay (#X6493, Thermo Fisher Scientific, Rockville, MD, USA) according to the manufacturer’s instructions, and the absorbances were measured by Synergy™ HTX Multi-Mode Microplate Reader with Gen5™ software (version 3.04, BioTek Instruments, Winooski, VT, USA). IC_50_ values were calculated using GraphPad Prism v. 7.1 (GraphPad Software, Inc., La Jolla, CA, USA). The degree of combination synergy, additivity, or antagonism was calculated using SynergyFinder (version R-3.10.3) [22] with a reference highest single agent (HSA) model. An HSA synergy score >10 indicates synergy, −10 to 10 suggests additivity and <−10 represents antagonism.

### 2.4. Clonogenic Assay

A total of 3000–20,000 cells were seeded in 12-well plates or 6-well plates and treated with ATRi, PI3Kαi, both or 0.01% DMSO after seeding. After 10 days, the media were aspirated, and colonies were fixed in methanol and stained with 0.01% crystal violet solution. Colony images were scanned and quantification of colony area percentage, more reflective of cell survival, was performed using Fiji software (version 2.17.0, NIH, Bethesda, MD, USA) with the ColonyArea Image J Plugin (version v1.0.1) [23].

### 2.5. Alkaline Comet Assay

The alkaline comet assay was performed to examine the DNA damage, as described before in [24]. Cells were collected after 48 h of drug treatment, followed by comet assay slide preparation. After the electrophoresis on day 1, the slides were later stained with SYBR Green dye for 5 min in the dark on day 2. Excess SYBR Green dye was removed after the staining, and the slides were washed 3 times with deionized water and dried in the dark overnight. The images were obtained using an ECLIPSE Ts2 microscope (Nikon, Melville, NY, USA). CometScore Pro (TriTrek Corporation, Sumerduck, VA, USA) was used to calculate the DNA damage index from the mean tail moment of three independent experiments (at least 100 cells were scored in each experiment).

### 2.6. Immunofluorescence Staining

γH2AX and RAD51 foci formation were examined by immunofluorescence staining. The cells were seeded and grown on ibidi^TM^ 8-well removable chamber slides (ibidi, Fitchburg, WI, USA) and treated with the indicated drugs for 48 h. Cells were then fixed in 4% paraformaldehyde for 10 min, permeabilized with 0.5% Triton-X 100 for another 10 min, followed by blocking in 5% bovine serum albumin for 1 h at 37 °C. The slides were then subjected to overnight incubation with fluorophore-conjugated antibodies (γH2AX, #613410, BioLegend, San Diego, CA, USA; RAD51, #ab309674, Abcam, Rockville, MD, USA) and were mounted with VECTASHIELD HardSet Antifade Mounting Medium with DAPI (#H-1500, Vector Laboratories, Burlingame, CA, USA) to stain the nucleus on the following day. Images were captured using a Leica Stellaris FLIM confocal microscope with a 63× oil-immersion objective. The signals were quantified using Fiji software (version 2.17.0) to evaluate the percentage of positive cells (cells with >5 foci).

### 2.7. S1 Nuclease DNA Fiber Assay

To measure the level of single-stranded DNA (ssDNA) gaps, the S1 nuclease DNA fiber assay was conducted as previously described [24]. Images were collected with Nikon SoRa Spinning Disk confocal microscope (Nikon, Tokyo, Japan) with a 60×/1.49 oil immersion objective. Fiber length was measured using ImageJ software (version 1.54g). At least 50 fibers are quantified for each experiment.

### 2.8. Immunoblotting

Western blot of proteins extracted from the cells was performed to validate the expression levels. Cell lysates were harvested with a scraper and protein was extracted with RIPA buffer (Invitrogen, Waltham, MA, USA) supplemented with Complete^®^ EDTA-free protease inhibitors and phosphatase inhibitors (MilliporeSigma, Rockville, MD, USA). Immunoblotting was then performed as previously described [25]. Images were taken and visualized by the Licor Odyssey Imaging System. The following antibodies were obtained from Cell Signaling Technology (Danvers, MA, USA): cleaved-PARP (c-PARP, #5625S), cleaved-caspase 3 (c-caspase 3, #9664S), GAPDH (#2118S), xCT (12691S), ECL goat anti-mouse IgG HRP (#7076), and ECL goat anti-rabbit IgG HRP (#7074).

### 2.9. Apoptotic Cell Analysis

Annexin V/Propidium Iodide (PI) staining was conducted to differentiate between early apoptotic, late apoptotic, and viable cells, as described elsewhere [26]. The samples were analyzed on a Sony ID 7000TM Spectral Cell Analyzer (Sony Biotechnology Inc., San Jose, CA, USA) and FlowJo software (version 10.7.2, FlowJo LLC, Ashland, OR, USA).

### 2.10. Cellular Ferrous Ion Detection

The levels of intracellular ferrous ions were assessed by FerroOrange probes (DOJINDO, Rockville, MD, USA) following the instructions provided by DOJINDO [27]. In brief, cells were seeded on ibidi^TM^ 8-well high glass bottom chamber slides (ibidi) and treated as indicated. Cells were then washed with HBSS, followed by 30 min of incubation with 1 μM FerroOrange at 37 °C, 5% CO_2_, in an incubator. Images were captured using a Leica Stellaris FLIM confocal microscope with a 20× objective. The mean fluorescence intensity was quantified using Fiji software.

### 2.11. GSH/GSSG-Glo^TM^ Assay

To detect total GSH and oxidized GSH (GSSG) in cultured cells, the GSH/GSSG-Glo^TM^ assay was conducted according to the manufacturer’s instructions, and luminescence was measured by a Synergy™ HTX Multi-Mode Microplate Reader with Gen5™ software.

### 2.12. Statistical Analysis

All statistical analyses were performed and calculated by GraphPad Prism version 10.2.0 (GraphPad Software, Boston, Massachusetts, USA). Student’s *t*-test or one-way ANOVA was used to determine statistical significance, and *p* < 0.05 was considered statistically significant. All data were repeated in triplicate and are shown as mean ± SD.

## 3. Results

### 3.1. Combined ATRi and PI3Kαi Treatment Demonstrates Additive/Synergistic Cytotoxicity Across EC Cell Lines Independent of Microsatellite Stability Status and Platinum Sensitivity

**Figure 1:**
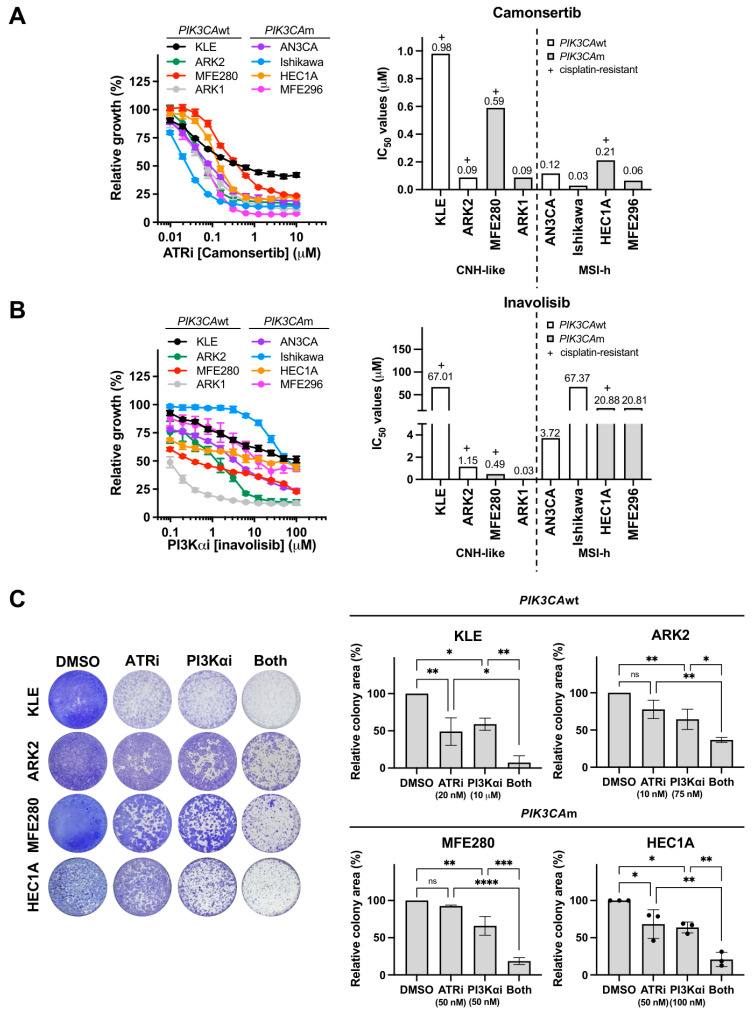
Single-agent and combined activity of ATRi camonsertib and PI3Kαi inavolisib in endometrial cancer cells. (**A**,**B**) Cell growth was measured by XTT assays. CNH-like and MSI-h EC cell lines were treated with the indicated concentrations of ATRi camonsertib (**A**) or PI3Kαi inavolisib (**B**) for 3 days and subjected to XTT assays. (**C**) Long-term survival was measured by colony-forming assay. Cells were treated with clinically attainable concentrations of ATRi camonsertib and PI3Kαi inavolisib and grown for 10 days. All experiments were repeated in triplicate. Data are shown as mean ± SD. ****, *p* < 0.0001; ***, *p* < 0.001; **, *p* < 0.01; *, *p* < 0.05; ns, not significant.

To investigate therapeutic strategies for platinum-resistant EC, we first assessed cisplatin sensitivity across eight EC cell lines representing diverse genetic backgrounds [28]. Four EC cell lines (KLE, MFE280, HEC1A, and ARK2) demonstrated resistance to clinically achievable concentrations of cisplatin [29] (IC_50_ 28.1–32.2 µM, Figure S1A). *PIK3CA* mutations were present in both cisplatin-sensitive and -resistant models (Figure S1A), consistent with their high prevalence in EC [15]. We then evaluated monotherapy responses to the ATRi camonsertib and PI3Kαi inavolisib using the same panel. All tested EC cell lines showed sensitivity to clinically attainable doses of ATRi (IC_50_ 0.03–0.98 µM, Figure 1A), while sensitivity to PI3Kαi varied substantially (IC_50_ 0.03–67.37 µM, Figure 1B), independent of their *PIK3CA* mutation status. This differential response highlighted the complexity of targeting these pathways individually and suggested that combination therapy might be necessary for optimal efficacy.

To explore this possibility, we performed 8 × 8 dose–response screens to assess the combinatorial effects of ATRi and PI3Kαi. The dual therapy exhibited robust additivity/synergy across all tested EC lines (HSA synergy scores −2.17 to 11.03; Figure S1B), using drug concentrations within the clinical range (camonsertib < 6 μM; inavolisib < 10 μM). Importantly, this synergistic cytotoxic effect was independent of microsatellite stability status and platinum sensitivity. Colony formation assays further confirmed that the combination significantly reduced long-term proliferation in both *PIK3CA*wt (KLE and ARK2) and *PIK3CA*m (MFE280 and HEC1A) platinum-resistant EC cells (Figure 1C). Collectively, these findings provide a rationale for targeting ATR and PI3K pathways in platinum-resistant EC.

### 3.2. Combined Inhibition of ATR and PI3Ka Pathways Induces DNA Damage in PIK3CAm but Not in PIK3Awt EC Cells

**Figure 2:**
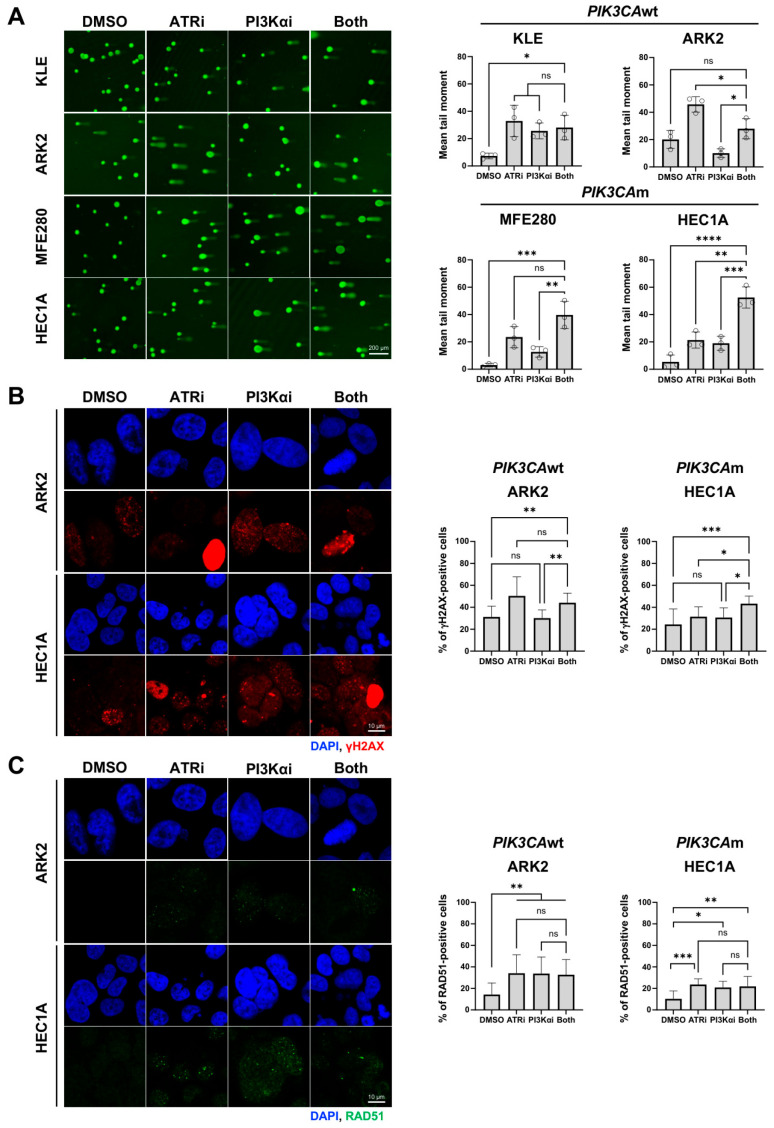
Dual inhibition of ATR and PI3Ka pathways induces greater DNA damage in *PIK3CA*m cells. (**A**) Cells were treated with ATRi and/or PI3Kαi, and their effect on DNA damage was measured by alkaline comet assay. The percentage of tail moment is plotted. (**B**,**C**) Immunofluorescence staining of γH2AX (**B**) and RAD51 (**C**) foci was performed on the same slide to examine DSBs and the functionality of homologous recombination repair, respectively. Cells with >5 foci were counted as positive cells. The percentage of γH2AX- or RAD51-positive cells is plotted. All experiments were repeated at least in triplicate. Data are shown as mean ± SD. ****, *p* < 0.0001; ***, *p* < 0.001; **, *p* < 0.01; * *p* < 0.05; ns, not significant.

To investigate whether the synergistic effect reflected enhanced genomic instability in platinum-resistant EC, we examined the DNA damage endpoints. Alkaline comet assays revealed significant increases in mean tail moment following combination treatment in *PIK3CA*m but not in *PIK3CA*wt cells (Figure 2A). Similarly, combined treatment significantly increased the γH2AX foci (double-stranded breaks [DSB] marker), which were markedly elevated only in *PIK3CA*m HEC1A (Figure 2B). However, RAD51 foci remained unchanged, suggesting that defective homologous recombination repair is unlikely to account for the synergistic cytotoxic effect (Figure 2C).

We next performed S1 nuclease-enhanced DNA fiber assays, as the accumulation of ssDNA gaps can act as a driver of replication stress and genomic instability [30]. Combination treatment showed minimal effects on replication tracks compared to monotherapy in both *PIK3CA*wt and *PIK3CA*m EC cell lines (Figure S2). Together, these findings indicate that the synergy of ATR and PI3K inhibition in *PIK3CA*m EC cells may be primarily mediated by DSB induction and genomic instability, rather than by replication-associated ssDNA gaps.

### 3.3. Co-Targeting ATR and PI3Ka Pathways Increases Apoptosis in PIK3CAm but Not in PIK3CAwt EC Cells

**Figure 3:**
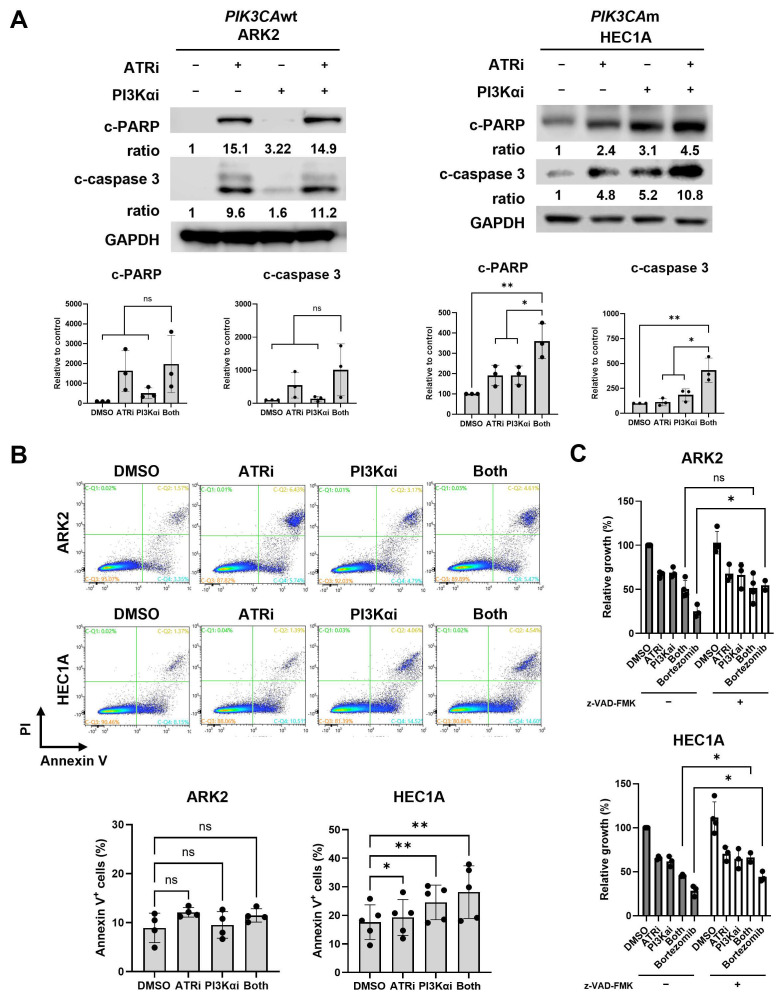
Combination treatment of ATRi and PI3Kai induces apoptosis in *PIK3CA*m cells. (**A**) Western blotting of c-PARP, c-caspase 3, and GAPDH. Densitometric values were normalized to the corresponding GAPDH levels. Protein expression is presented as a percentage relative to the control group. (**B**) PI-Annexin V staining cells were analyzed by flow cytometry. The percentage of early apoptotic Annexin V-positive (Annexin V^+^) cells is plotted. (**C**) Cell growth was measured by XTT assays. EC cell lines were treated with a pan-caspase inhibitor z-VAD-FMK for 2 h before being treated with ATRi camonsertib and/or PI3Kαi inavolisib and grown for 3 days. Bortezomib, a proteosome inhibitor and apoptosis inducer, was used as a positive control. All experiments were repeated at least in triplicate. Data are shown as mean ± SD. **, *p* < 0.01; * *p* < 0.05; ns, not significant. The uncropped blots are shown in Supplementary S2 file.

We then examined apoptosis induction by immunoblotting and Annexin V staining. In *PIK3CA*m HEC1A cells, combination therapy induced greater levels of c-PARP and c-caspase 3 and led to a higher proportion of Annexin V-positive cells (Figure 3A,B). Pre-treatment with pan-caspase inhibitor z-VAD-FMK rescued cell viability in HEC1A but had no significant effect on *PIK3CA*wt ARK2 cells (Figure 3C). These results suggest that the combination may promote apoptosis via DSB accumulation in *PIK3CA*m but induce cell death through alternate mechanisms in *PIK3CA*wt cells.

### 3.4. Dual Inhibition of ATR and PI3Ka Pathways Enhances Ferroptosis in PIK3CAwt but Not in PIK3CAm EC Cells

**Figure 4:**
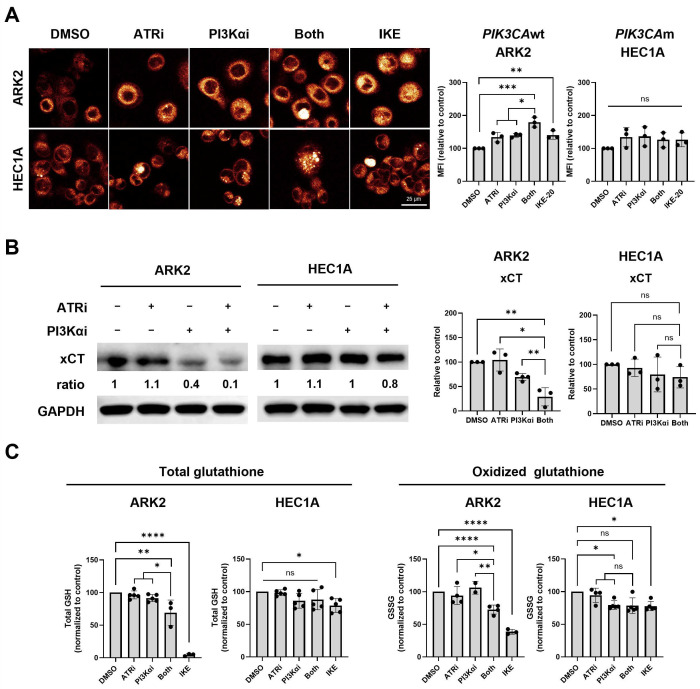
Combined ATRi and PI3Kai treatment induces ferroptosis in *PIK3CA*wt cells via downregulating xCT. (**A**) The levels of cellular ferrous ion were assessed by FerroOrange probe labeling. The mean FerroOrange fluorescence intensity (MFI) of each cell was quantified, and the values of each group relative to the control are shown. (**B**) Western blotting of xCT and GAPDH. Densitometric values were normalized to the corresponding GAPDH levels. Protein expression is presented as a percentage relative to the control group. (**C**) Intracellular levels of GSH and GSSG in EC cells were assessed by the GSH/GSSG-Glo assay. All experiments were repeated at least in triplicate. Data are shown as mean ± SD. ****, *p* < 0.0001; ***, *p* < 0.001; **, *p* < 0.01; * *p* < 0.05; ns, not significant. The uncropped blots are shown in Supplementary S2 file.

Because *PIK3CA*wt cells showed minimal DNA damage or apoptosis under combination treatment, we investigated ferroptosis as an alternative death mechanism [8,31]. FerroOrange staining demonstrated significantly elevated intracellular ferrous iron in *PIK3CA*wt ARK2 but not in *PIK3CA*m HEC1A cells after combination treatment (Figure 4A). In ARK2 cells, immunoblotting further confirmed that combination therapy suppressed the cystine/glutamate antiporter xCT, which protects against lipid peroxidation (Figure 4B). In contrast, HEC1A cells showed no reduction in xCT (Figure 4B). Pre-treatment with ferroptosis inhibitor ferrostatin-1 significantly rescued cell death in ARK2 but showed no rescue in HEC1A cells (Figure S3A), indicating that ferroptosis is the predominant mode of cell death induced by the combination treatment in ARK2 cells.

To further evaluate redox homeostasis, we measured total GSH and GSSG levels using the GSH/GSSG-Glo assay. Consistent with the above findings, combination therapy significantly reduced both total GSH and GSSG levels and redox capacity in ARK2 but only a minimal decrease in HEC1A cells (Figure 4C). As a ferroptosis inducer, IKE decreased GSH and GSSG levels in ARK2 cells but exerted modest effects in HEC1A cells (Figure 4C). Despite the reduction in total GSH levels, no significant differences in the GSH/GSSG ratio were observed among treatment groups, suggesting an overall depletion of the GSH pool rather than a shift in redox balance (Figure S3B). Collectively, these findings support a ferroptosis-associated redox alteration in *PIK3CAwt* ARK2 cells following dual ATR and PI3Kα inhibition.

### 3.5. Combined Inhibition of ATR and PI3Ka Suppresses PIK3CAwt Patient-Derived Cells via Ferroptosis Induction

**Figure 5:**
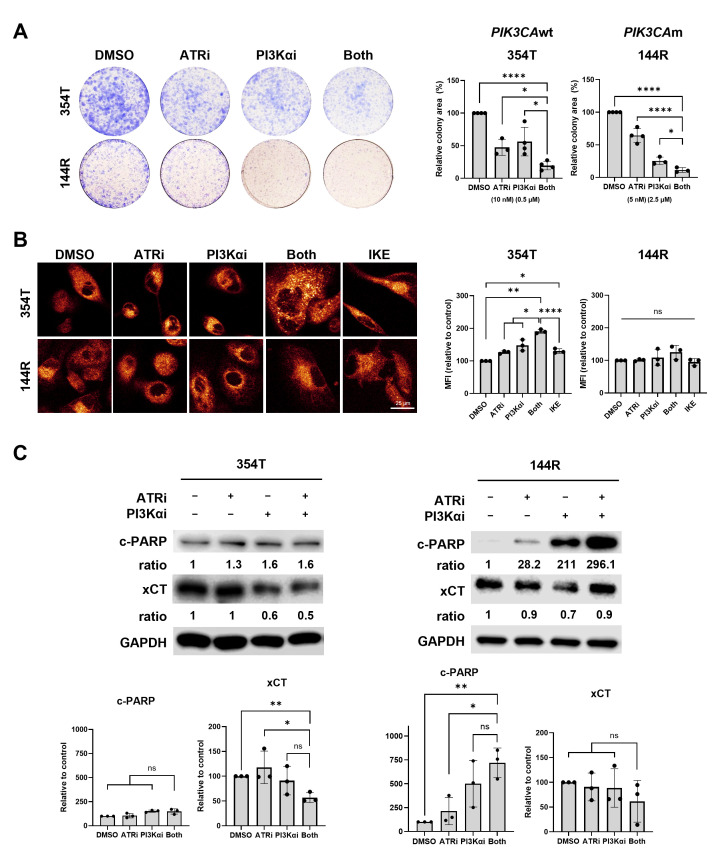
Combined inhibition of ATR and PI3Kα induces ferroptosis in *PIK3CA*wt patient-derived cell line. (**A**) Colony-forming assay was used to measure the long-term survival of EC PDCs upon treatment with clinically attainable concentrations of ATRi camonsertib and PI3Kαi inavolisib for 10 days. (**B**) The levels of cellular ferrous ion were assessed by FerroOrange probe labeling. The mean fluorescence intensity (MFI) of each cell line was quantified, and the values of each group relative to the control are shown. (**C**) Western blotting of c-PARP, xCT, and GAPDH. Densitometric values were normalized to the corresponding GAPDH levels. Protein expression is presented as a percentage relative to the control group. All experiments were repeated at least in triplicate. Data are shown as mean ± SD. ****, *p* < 0.0001; **, *p* < 0.01; *, *p* < 0.05; ns, not significant. The uncropped blots are shown in Supplementary S2 file.

Lastly, to validate the clinical relevance of our findings, we evaluated two serous EC PDC lines, *PIK3CA*wt platinum-resistant 354T and *PIK3CA*m treatment-naïve 144R, which have never received prior platinum-based chemotherapy. Both PDC lines showed reduced cell proliferation with the combination treatment (Figure 5A). However, ferroptosis markers including increased intracellular iron (Figure 5B) and downregulation of xCT (Figure 5C), were observed only in *PIK3CA*wt 354T but not in *PIK3CA*m 144R cells. Similarly, IKE treatment only increased intracellular iron levels in 354T cells but not in 144R cells (Figure 5C). Taken together, these results support a model in which dual ATR and PI3Kα inhibition induces ferroptosis selectively in *PIK3CA*wt EC cells, providing a rationale for genotype-based therapeutic stratification in recurrent EC.

## 4. Discussion

Platinum resistance remains a critical therapeutic challenge in EC, with limited options once standard chemotherapy fails [32]. Notably, the PI3K pathway is among the most frequently altered signaling cascades in EC [15], and its constitutive activation promotes replication stress and metabolic reprogramming that may influence therapeutic response. While inhibitors of ATR (e.g., camonsertib, berzosertib) and PI3Kα (e.g., alpelisib, serabelisib) have shown promise in preclinical studies, most efforts have focused on reactivating apoptosis [33,34,35,36,37,38]. However, platinum-resistant tumors frequently bypass apoptotic mechanisms, highlighting the need for alternative cell death pathways. Ferroptosis, which relies on iron-dependent lipid peroxidation to trigger cell death, presents a viable strategy to bypass apoptosis resistance [31]. Supporting this, recent transcriptomic analyses reveal that low ferroptosis pathway activity correlates with relapse after platinum- or taxane-based therapy in EC [39,40], highlighting the need for therapeutic strategies that can reactivate this pathway. Our study addresses this gap by demonstrating that dual inhibition of ATR and PI3Kα induces *PIK3CA* genotype-specific cell death: apoptosis in *PIK3CA*m EC cells and predominantly ferroptosis in *PIK3CA*wt EC cells. This dual mechanism uncovers a new therapeutic vulnerability and highlights *PIK3CA* mutation status as a clinically actionable biomarker.

Mechanistically, in *PIK3CA*m EC models, dual ATR and PI3Kα inhibition induces extensive DNA damage and apoptosis. This synthetic lethality can be explained by the convergence of two vulnerabilities: First, *PIK3CA* mutations compromise the DDR through constitutive PI3K/AKT signaling, which promotes genomic instability and impairs DNA repair pathways [41,42,43]. For instance, Juvekar et al. demonstrated that PI3K inhibitors selectively trigger DNA damage in DDR-deficient tumors [42], and Brown et al. identified MERIT40 as an AKT substrate essential for resolving replication stress [43]. Second, ATR is a critical kinase for managing replication stress [17]. When both pathways are inhibited simultaneously, these DDR-compromised *PIK3CA*m EC cells become unable to manage the accumulated replication stress, leading to catastrophic DNA damage and apoptosis.

In contrast to the apoptotic response in *PIK3CA*m cells, *PIK3CA*wt EC models predominantly underwent ferroptosis in response to the same dual inhibition. This differential response can be explained by distinct signaling contexts: PI3K/AKT signaling regulates antioxidant and lipid metabolism pathways [44,45,46], and its inhibition reduces antioxidant capacity, rendering cells vulnerable to oxidative lipid damage. In *PIK3CA*wt cells, where PI3K signaling is less constitutively active, dual inhibition can more effectively suppress these protective mechanisms. ATRi may further sensitize cells to ferroptosis by disrupting DDR-ferroptosis crosstalk. Specifically, ATR stabilizes the deubiquitinase USP20, which promotes ferroptosis resistance [18], and ATR inhibition has been shown to induce ferroptosis in erythroblasts [21]. Together, these mechanisms likely cooperate to drive ferroptotic cell death in *PIK3CA*wt EC, where apoptotic pathways appear less dominant.

Notably, ferroptosis induction was ineffective in *PIK3CA*m models despite the use of the same treatment regimen. This resistance likely stems from the same constitutive PI3K/AKT activation that confers DDR deficiency. Specifically, aberrant AKT signaling enhances antioxidant defenses and reprograms lipid metabolism [47,48,49], effectively buffering against ferroptotic stimuli. Beyond this general mechanism, co-occurring genomic alterations may provide additional layers of resistance. For example, the *PPP2R1A* W257L loss-of-function mutation identified in HEC1A cells [50] may contribute to ferroptosis resistance. *PPP2R1A* encodes the scaffold A subunit of the protein phosphatase 2A (PP2A), which regulates ferroptosis via the PP2A/AMP-activated protein kinase axis [51,52]. Loss of PP2A function would be expected to further impair ferroptosis induction. These findings suggest that overcoming ferroptosis resistance in *PIK3CA*m EC may require co-targeting both PI3K/AKT-mediated antioxidant pathways and PP2A-related signaling cascades.

This genotype-specific ferroptosis response is supported by findings in other cancer types. AKT suppression sensitizes ovarian, lung, and osteosarcoma cells to cisplatin-induced ferroptosis [53,54,55]. In colorectal cancer, where the majority of tumors are *PIK3CA*wt [56], PI3K/AKT inhibition activates ferroptosis through the FTO/YTHDF2/GPX4 axis [57]. Similarly, in multiple myeloma, which also typically lacks *PIK3CA* mutations [58], the AP-1 inhibitor T-5224 induces ferroptosis by targeting PI3K/AKT signaling [59]. These observations suggest a broader applicability of PI3K/AKT-targeted ferroptosis induction in *PIK3CA*wt tumors across cancer types.

From a translational perspective, our results indicate that *PIK3CA* mutation status and ferroptosis regulators such as xCT may serve as predictive biomarkers to guide patient selection for ATRi and PI3Kαi combination therapy. Several ongoing clinical trials are evaluating PI3K inhibitors (e.g., inavolisib in *PIK3CA*m breast cancer [60]; alpelisib in *PIK3CA*m recurrent EC [61]) and ATRis in combination with DDR inhibitors in recurrent gynecologic cancers [62]. Incorporating ferroptosis-related biomarkers into these trials may enable rational patient stratification and enhance therapeutic efficacy.

Some limitations of this study should be acknowledged. Although ferroptosis induction was supported by increased intracellular iron levels and rescue by ferrostatin-1, direct detection of lipid peroxidation (e.g., malondialdehyde or C11-BODIPY assays) was not performed under our experimental conditions. Therefore, further mechanistic studies will be required to definitively confirm lipid peroxidation as the terminal execution event. In addition, our conclusions are based on a limited number of *PIK3CA*wt and *PIK3CA*m cell lines and patient-derived models. Although *PIK3CA* mutations are often associated with sensitivity to PI3Kα inhibitors [63], our findings suggest that therapeutic response may also be influenced by additional genomic contexts within the PI3K pathway, such as upstream HER2 activation, co-occurring mutations affecting chromatin remodeling (ARID1A loss [64]), or *PTEN* mutation status. Thus, validation in a larger panel of genetically diverse EC models and in vivo models will be necessary to strengthen the generalizability and translational relevance of dual ATR and PI3Kα inhibition.

## 5. Conclusions

In summary, our study reveals that *PIK3CA* mutation status may influence the cell death modality in EC following dual ATR and PI3Kα inhibition. In *PIK3CA*m cells, this combination induces apoptosis through DNA damage accumulation, whereas in *PIK3CA*wt cells, it predominantly triggers ferroptosis via antioxidant pathway suppression. These findings establish a genotype-informed therapeutic strategy for platinum-resistant EC and identify *PIK3CA* mutation status as a predictive biomarker.

## Data Availability

The original contributions presented in this study are included in the article/Supplementary Material. Further inquiries can be directed to the corresponding author.

## Supplementary Materials

The following supporting information can be downloaded at https://www.mdpi.com/article/10.3390/cancers18071064/s1. Figure S1: IC_50_ determination for cisplatin in endometrial cancer cell lines and synergy analysis of inavolisib and camonsertib; Figure S2: PI3Kαi enhances ATRi-induced ssDNA gap formation in *PIK3CA*wt ARK2 cells but not in *PIK3CA*m HEC1A cells; Figure S3: Rescue of ferroptosis and assessment of redox homeostasis. The uncropped blots are shown in Supplementary S2 file.

## Abbreviations

The following abbreviations are used in this manuscript:

AKT	Protein kinase B
ATR	Ataxia telangiectasia and Rad3-related kinase
ATRi	ATR inhibitor
c-caspase 3	Cleaved caspase 3
CNH	Copy number high
c-PARP	Cleaved poly (ADP-ribose) polymerase
DDR	DNA damage response
DSB	DNA double-stranded breaks
EC	Endometrial cancer
FTO	Fat mass and obesity-associated protein
YTHDF2	YTH N6-methyladenosine RNA binding protein F2
GPX4	Glutathione peroxidase 4
GSH	Glutathione
GSSG	Oxidized glutathione
HSA	Highest single agent
MFI	Mean fluorescence intensity
MSI-h	Microsatellite instability-high
PDC	Patient-derived cell
PI3K	Phosphatidylinositol 3-kinase
PI3Kα	Phosphatidylinositol-4,5-bisphosphate 3-kinase catalytic subunit alpha
PI3Kαi	PI3Kα inhibitor
*PIK3CA*m	*PIK3CA*-mutant
*PIK3CA*wt	*PIK3CA*-wildtype
PI	Propidium Iodide
PP2A	Protein phosphatase 2A
ssDNA	Single-stranded DNA
USP20	Ubiquitin-specific peptidase 20
xCT	Glutamate/cystine antiporter solute carrier family 7 member 11
