# Supplementary material for: Targeting ATR and PI3Kα Pathways Promotes Ferroptosis in PIK3CA-Wildtype Platinum-Resistant Endometrial Cancer
Source: Cancers (Basel). 2026 Mar 25;18(7):1064. doi: 10.3390/cancers18071064 (PMC13071977; doi:10.3390/cancers18071064)
Supplement: Supplementary file 1 [file cancers-18-01064-s001.zip › cancers-4189632-S1.pdf]

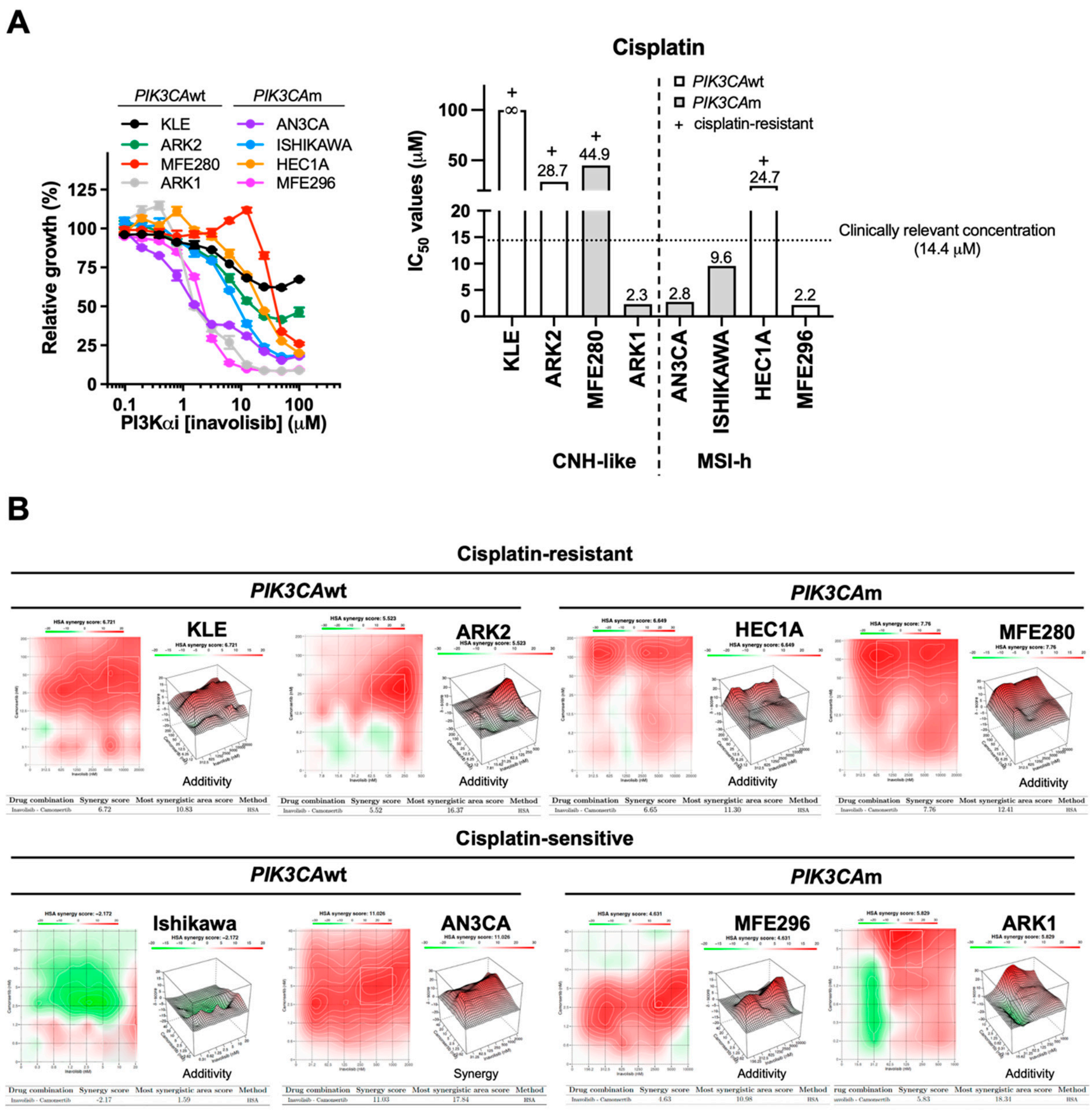

Figure S1. IC_{50} determination for cisplatin in endometrial cancer cell lines and synergy analysis of inavolisib and camonsertib. (A) Cell viability was assessed using XTT assays. Endometrial cancer (EC) cell lines were treated with the indicated concentrations of cisplatin for 3 days, followed by XTT analysis to determine IC50 values. (B) Cisplatin-sensitive microsatellite instability–high (MSI-H) EC cell lines (Ishikawa, AN3CA, MFE296) and copy number high (CNH)-like EC cell lines—including cisplatin-resistant (KLE, ARK2, HEC1A, MFE280) and cisplatin-sensitive (ARK1) lines—were evaluated for response to the ATR inhibitor (ATRi) camonsertib and the PI3Kα inhibitor (PI3Kαi) inavolisib. Cell viability was measured after 3 days using XTT assays. Drug interaction effects (synergy, additivity, or antagonism) were calculated using SynergyFinder based on the Highest Single Agent (HSA) reference model. An HSA synergy score >10 indicates synergy, −10 to 10 indicates additivity, and <−10 indicates antagonism. Experiments were performed in triplicate.

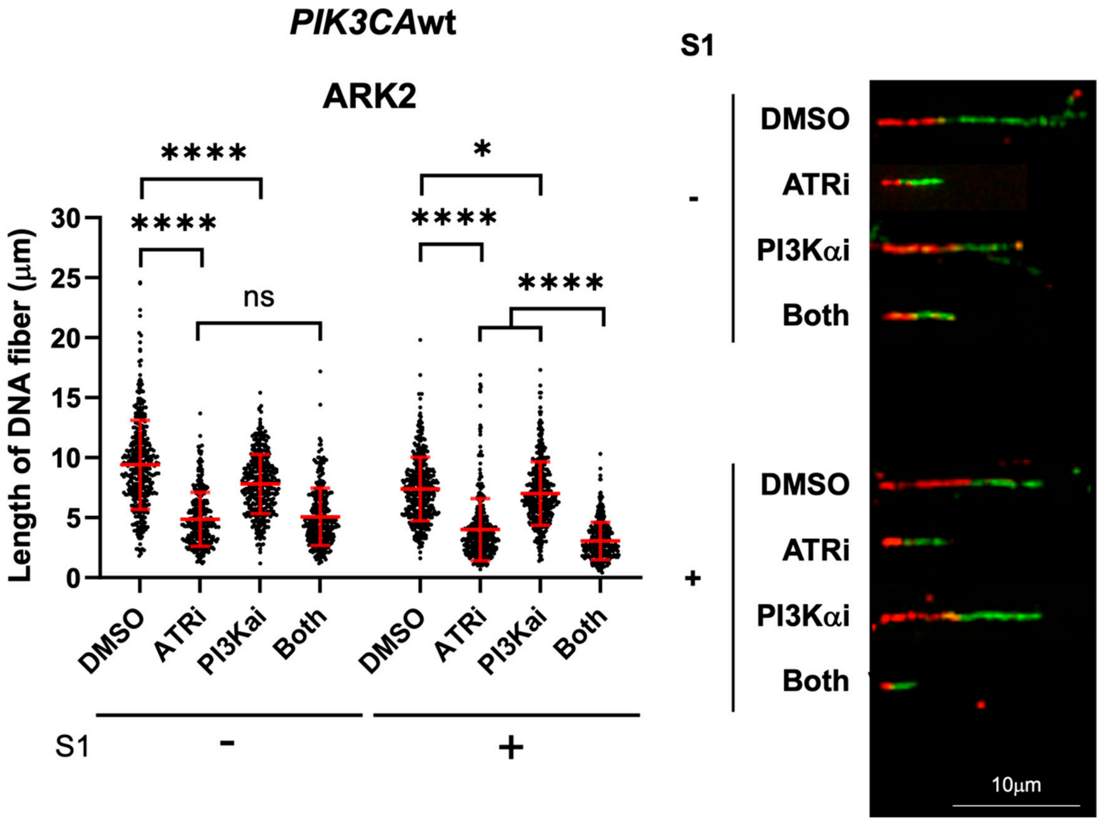
PIK3CAwt
ARK2
Length of DNA fiber (µm)
30
25
20
15
10
5
0
****
****
ns
*
****
****
DMSO
ATRi
PI3Kai
Both
DMSO
ATRi
PI3Kai
Both
S1
-
+
S1
-
DMSO
ATRi
PI3Kαi
Both
+
DMSO
ATRi
PI3Kαi
Both
10µm

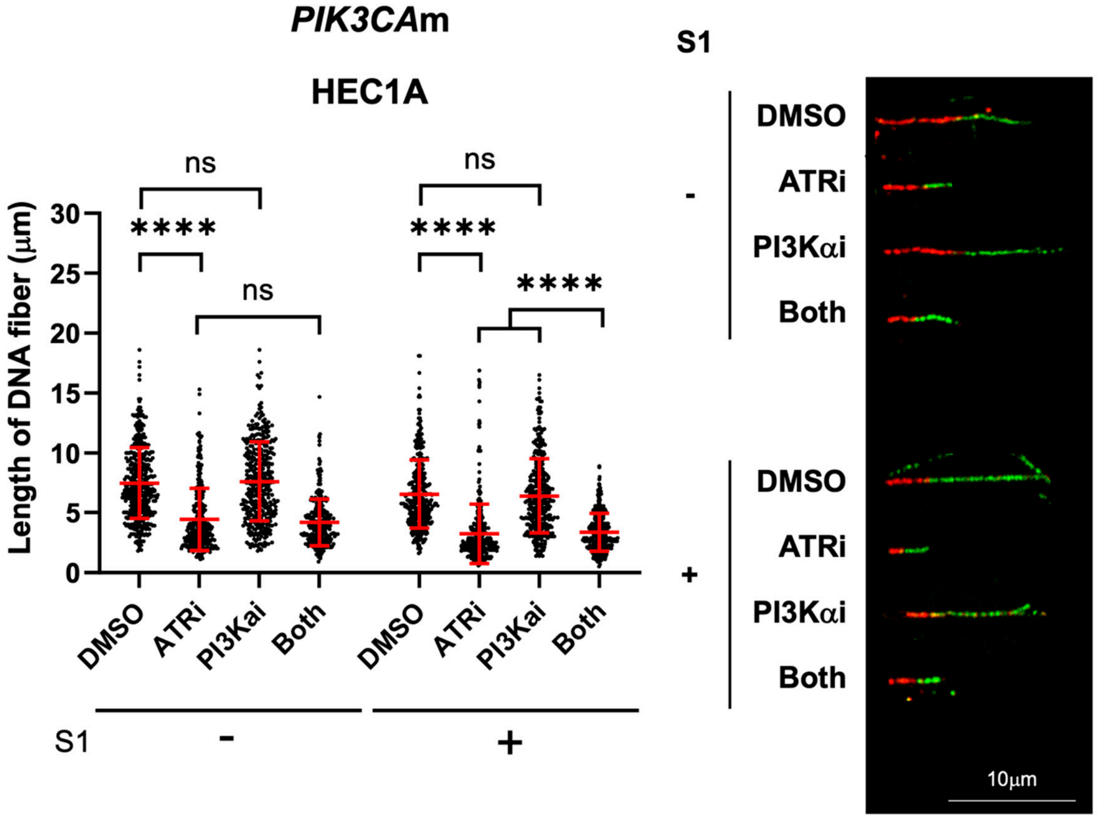
PIK3CAm
HEC1A
Length of DNA fiber (µm)
30
25
20
15
10
5
0
ns
****
ns
ns
****
****
DMSO
ATRi
PI3Kai
Both
DMSO
ATRi
PI3Kai
Both
S1
-
+
S1
-
DMSO
ATRi
PI3Kαi
Both
+
DMSO
ATRi
PI3Kαi
Both
10µm

Figure S2. PI3Kαi enhances ATRi-induced ssDNA gap formation in *PIK3CA*wt ARK2 cells but not in *PIK3CA*m HEC1A cells. DNA fiber assays were performed in ARK2 and HEC1A cells using sequential CldU and IdU labeling of nascent DNA tracts, with or without S1 nuclease treatment. Cells were exposed to the indicated treatments for 2 hours, followed by thymidine analog incorporation and subsequent S1 nuclease incubation to assess ssDNA gap formation. All experiments were conducted in triplicate. Data are presented as mean ± SD. ****, $p < 0.0001$; * $p < 0.05$; ns, not significant.

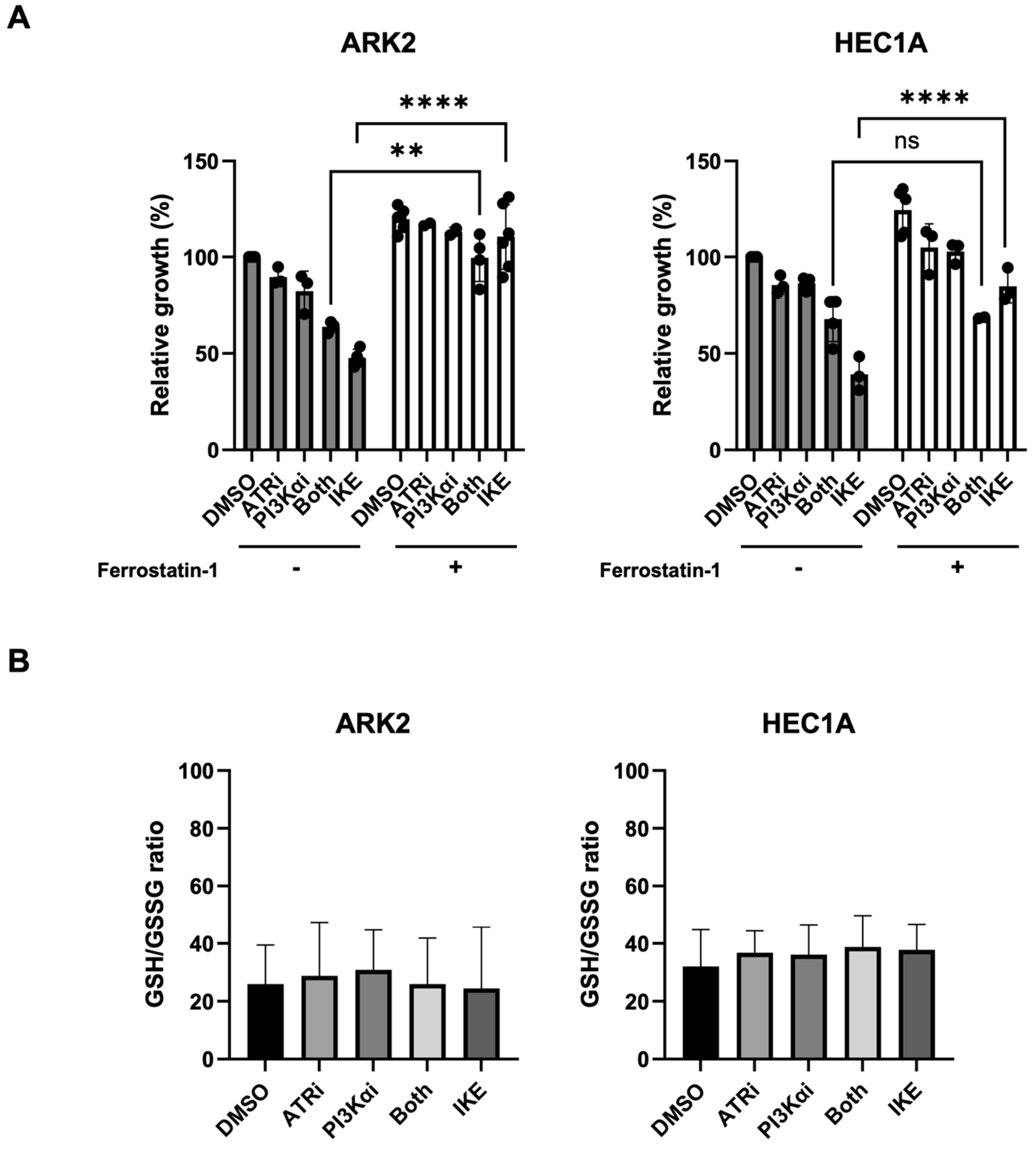

Figure S3. Rescue of ferroptosis and assessment of redox homeostasis. (A) Cell viability was evaluated using XTT assays. Cells were pretreated with the ferroptosis inhibitor ferrostatin-1 (20 μM) for 2 hours prior to treatment with the ATR inhibitor (ATRi) camonsertib and/or the PI3Kα inhibitor (PI3Kαi) inavolisib, followed by 3 days of growth. Imidazole ketone erastin (IKE), a system xc^{-} inhibitor and ferroptosis inducer, served as a positive control. (B) Intracellular redox status was assessed by measuring the ratio of reduced glutathione (GSH) to oxidized glutathione (GSSG) using the GSH/GSSG-Glo assay in EC cells. ****, $p < 0.0001$; **, $p < 0.01$; ns, not significant.
