# Supplementary material for: Targeting ATR and PI3Kα Pathways Promotes Ferroptosis in PIK3CA-Wildtype Platinum-Resistant Endometrial Cancer
Source: Cancers (Basel). 2026 Mar 25;18(7):1064. doi: 10.3390/cancers18071064 (PMC13071977; doi:10.3390/cancers18071064)

Fig. 2B

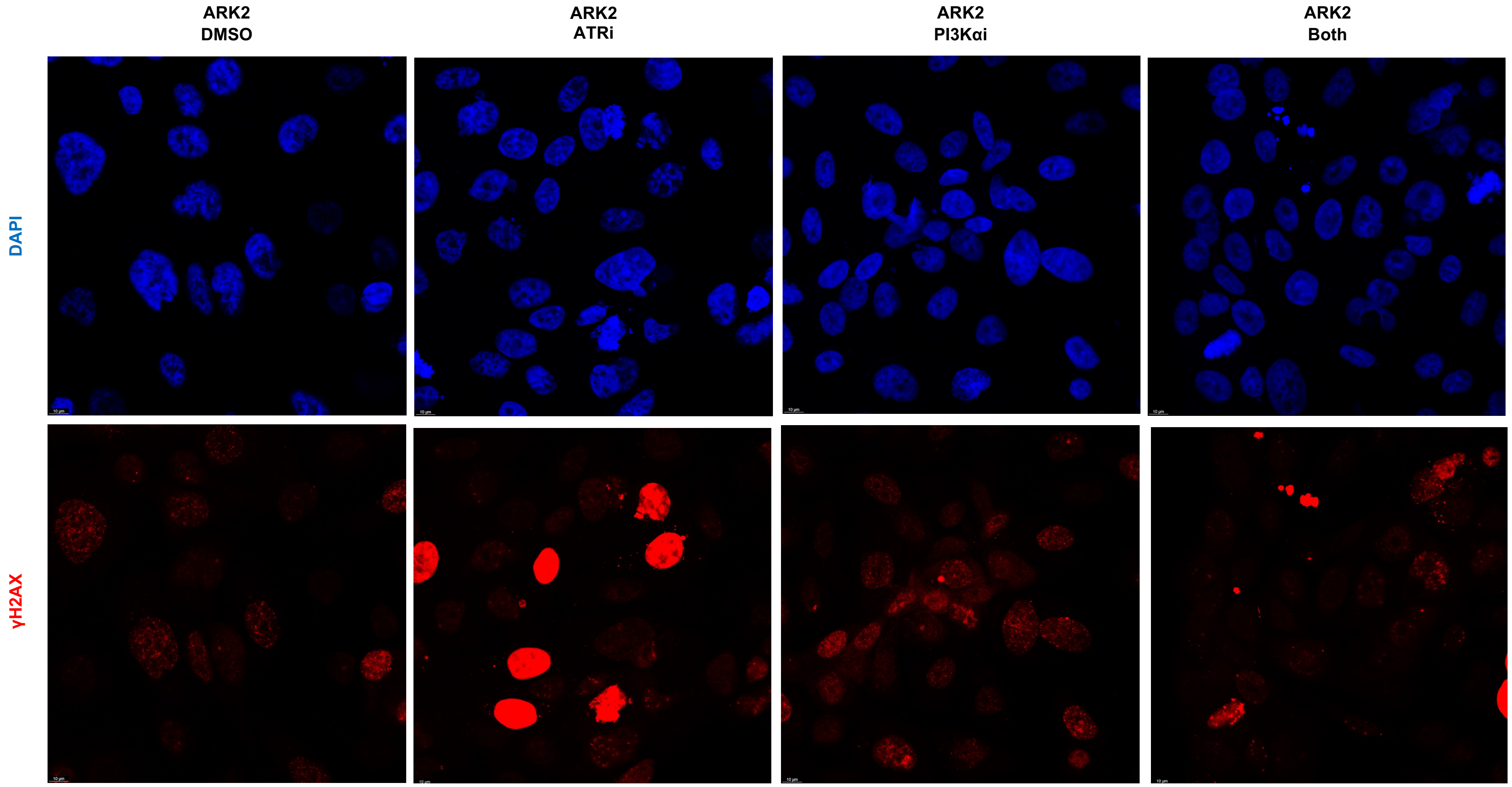

Fig. 2B

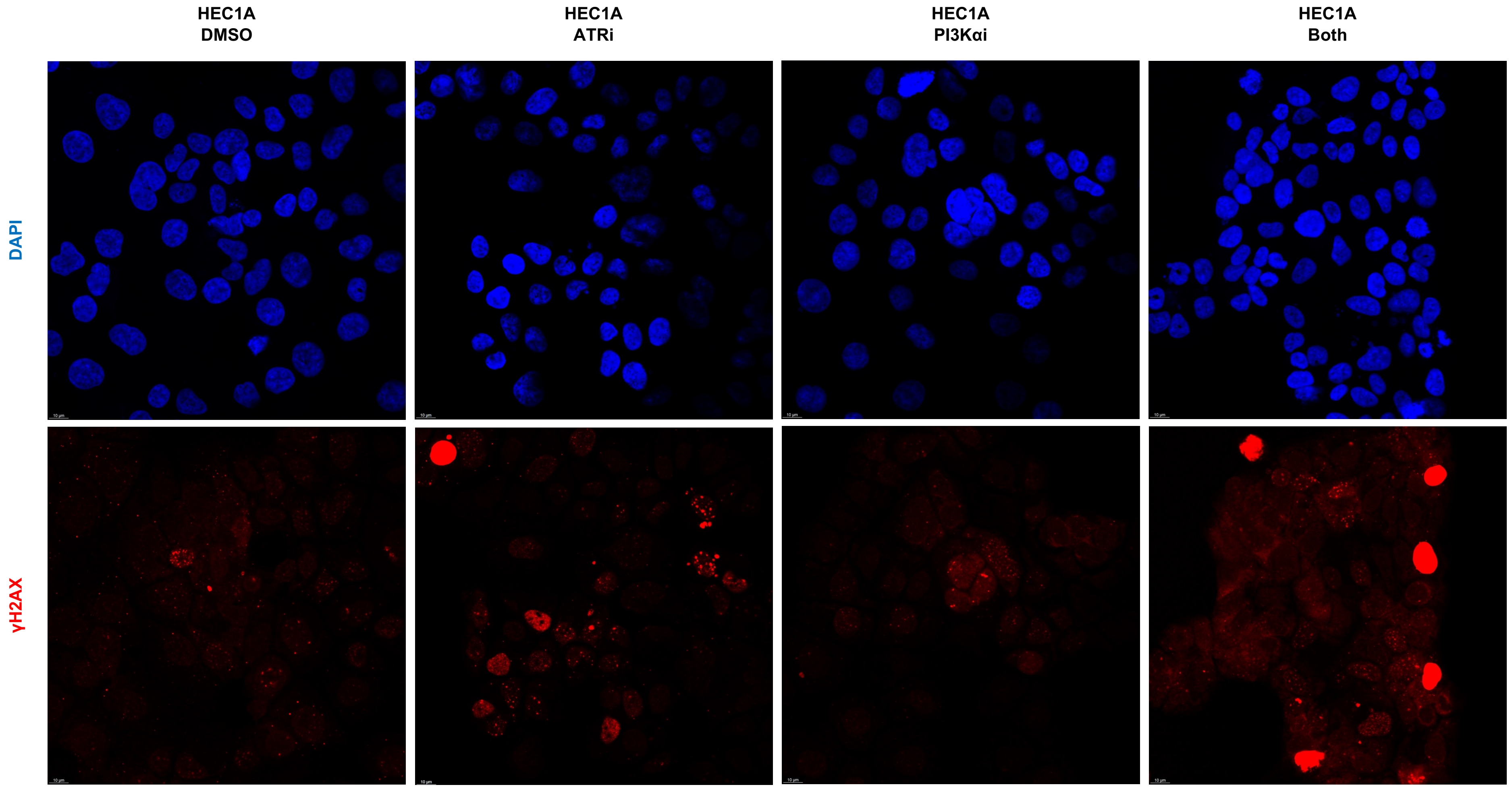

Fig. 2C

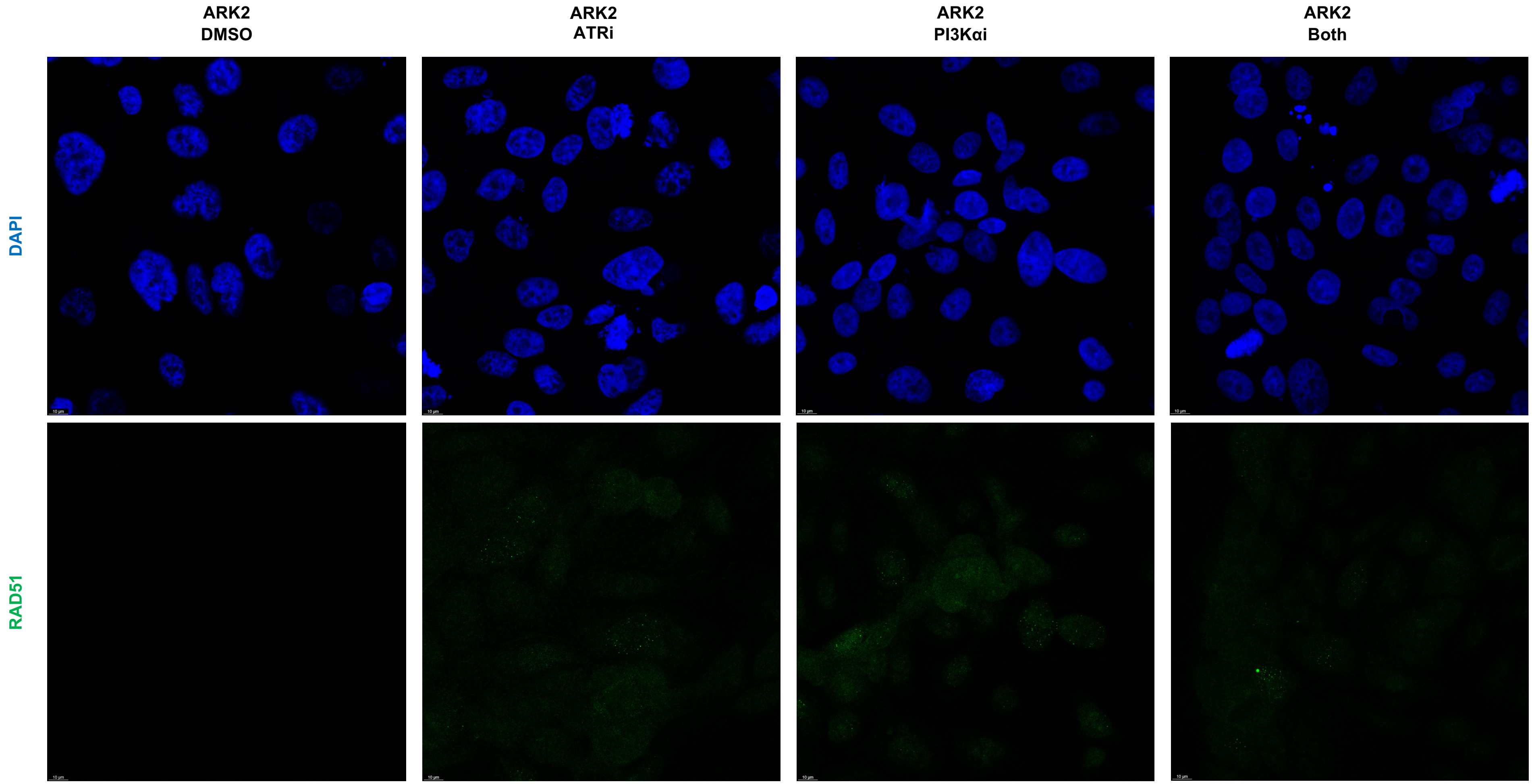

Fig. 2C

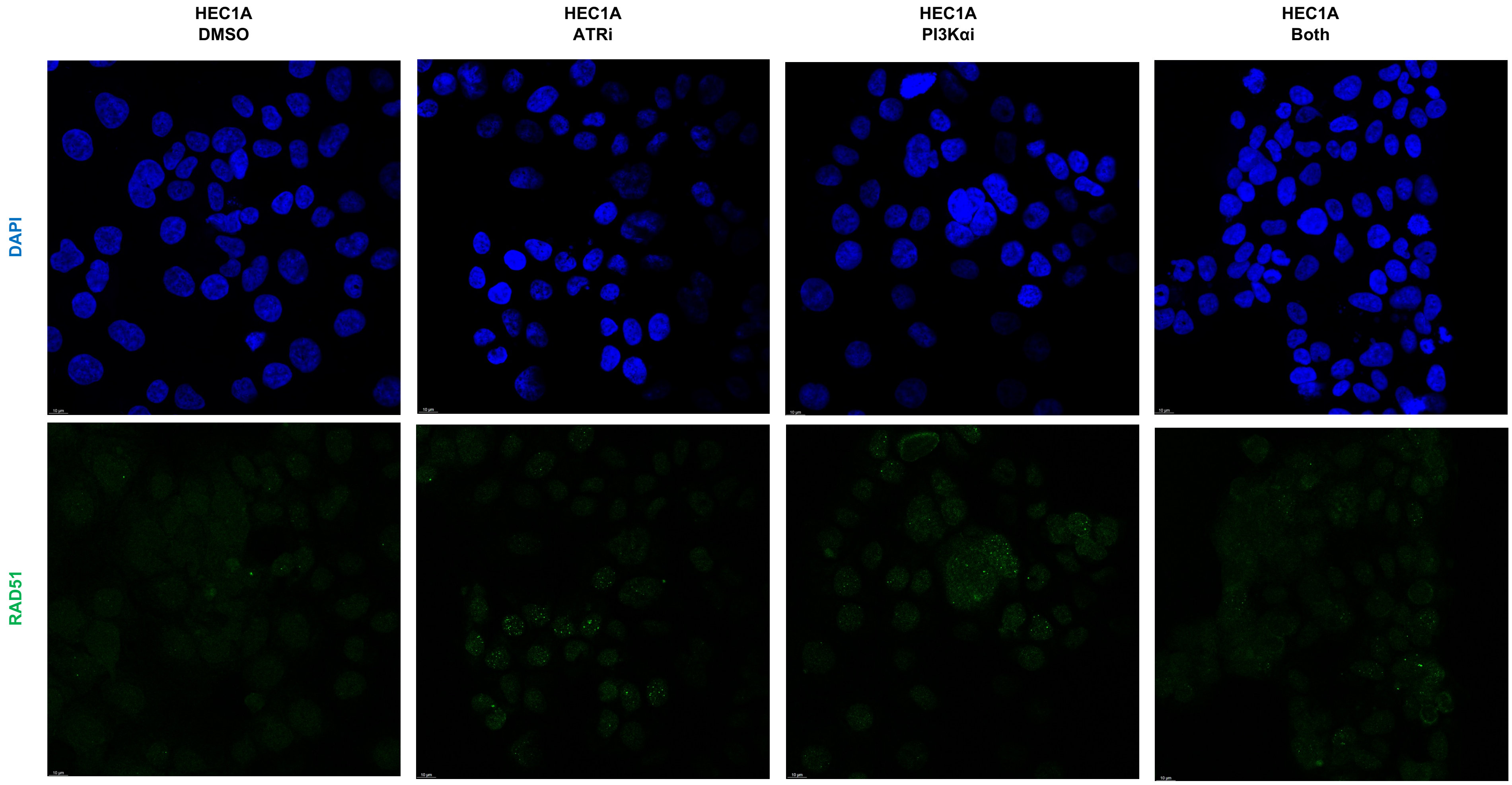

Fig. 3A

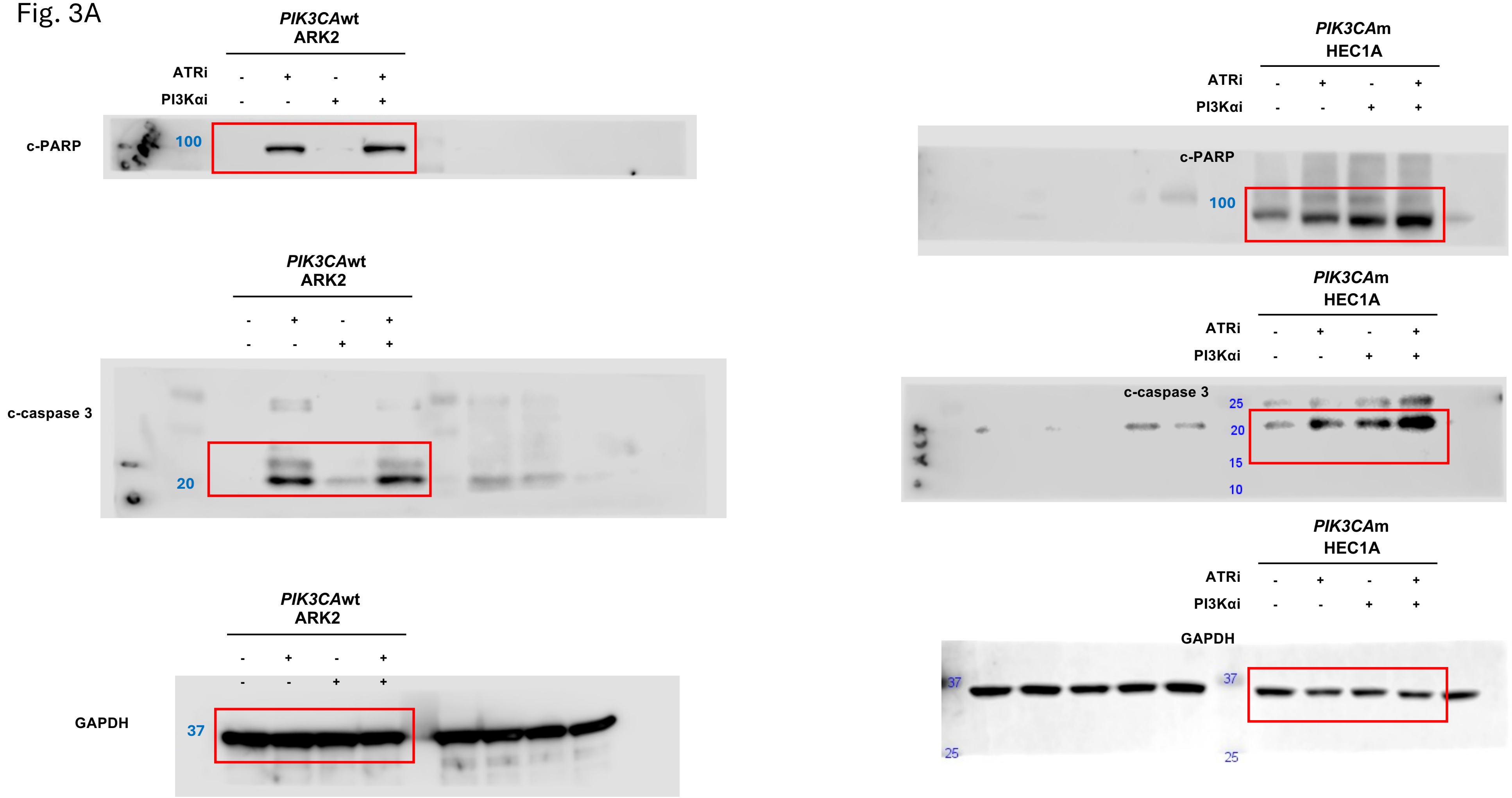

PIK3CAwt
ARK2
ATRi
PI3Kαi
c-PARP
100
c-caspase 3
20
GAPDH
37
PIK3CAm
HEC1A
c-PARP
100
c-caspase 3
25
20
15
10
GAPDH
37
25

Fig. 4A

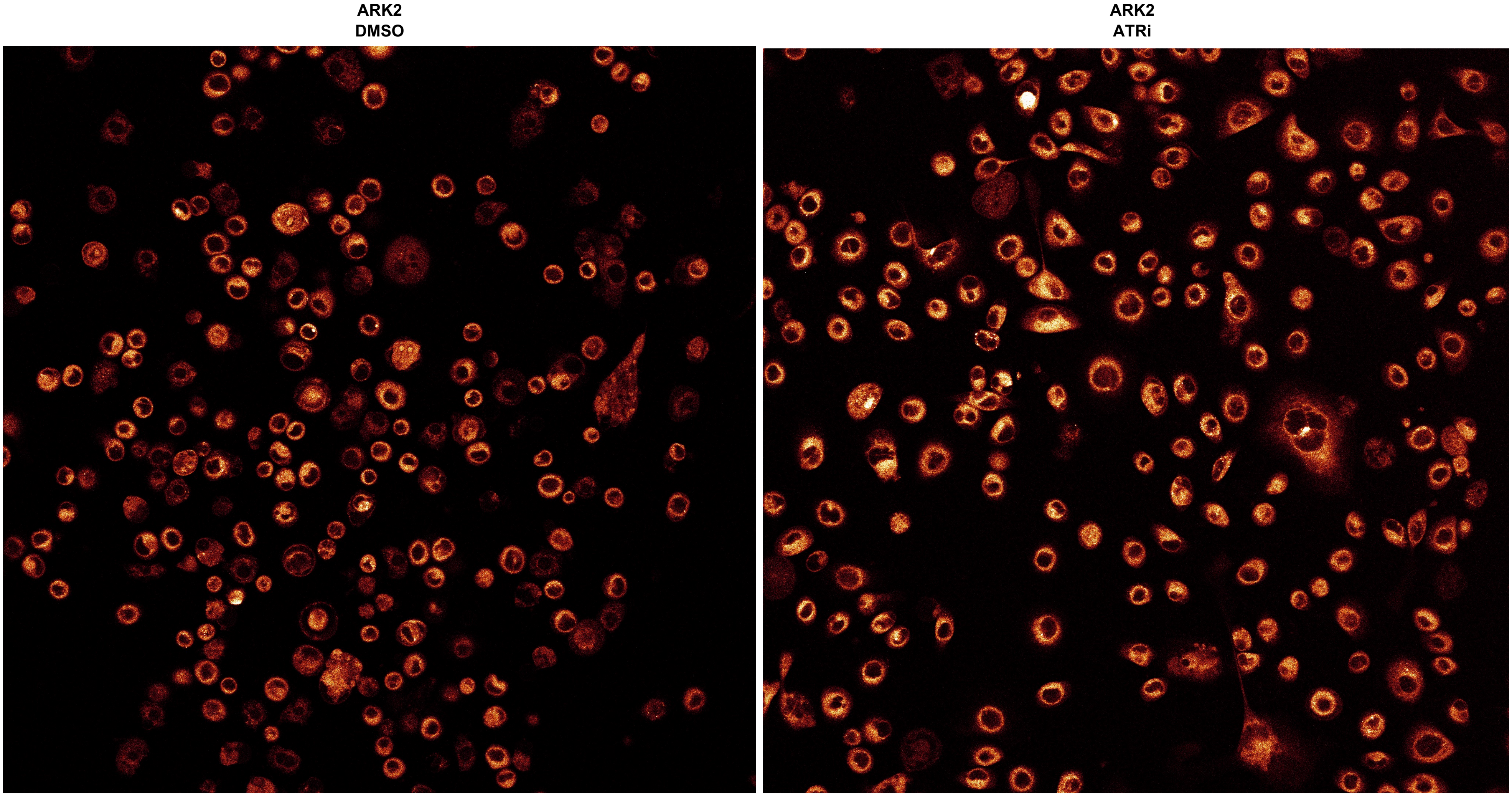

Fig. 4A

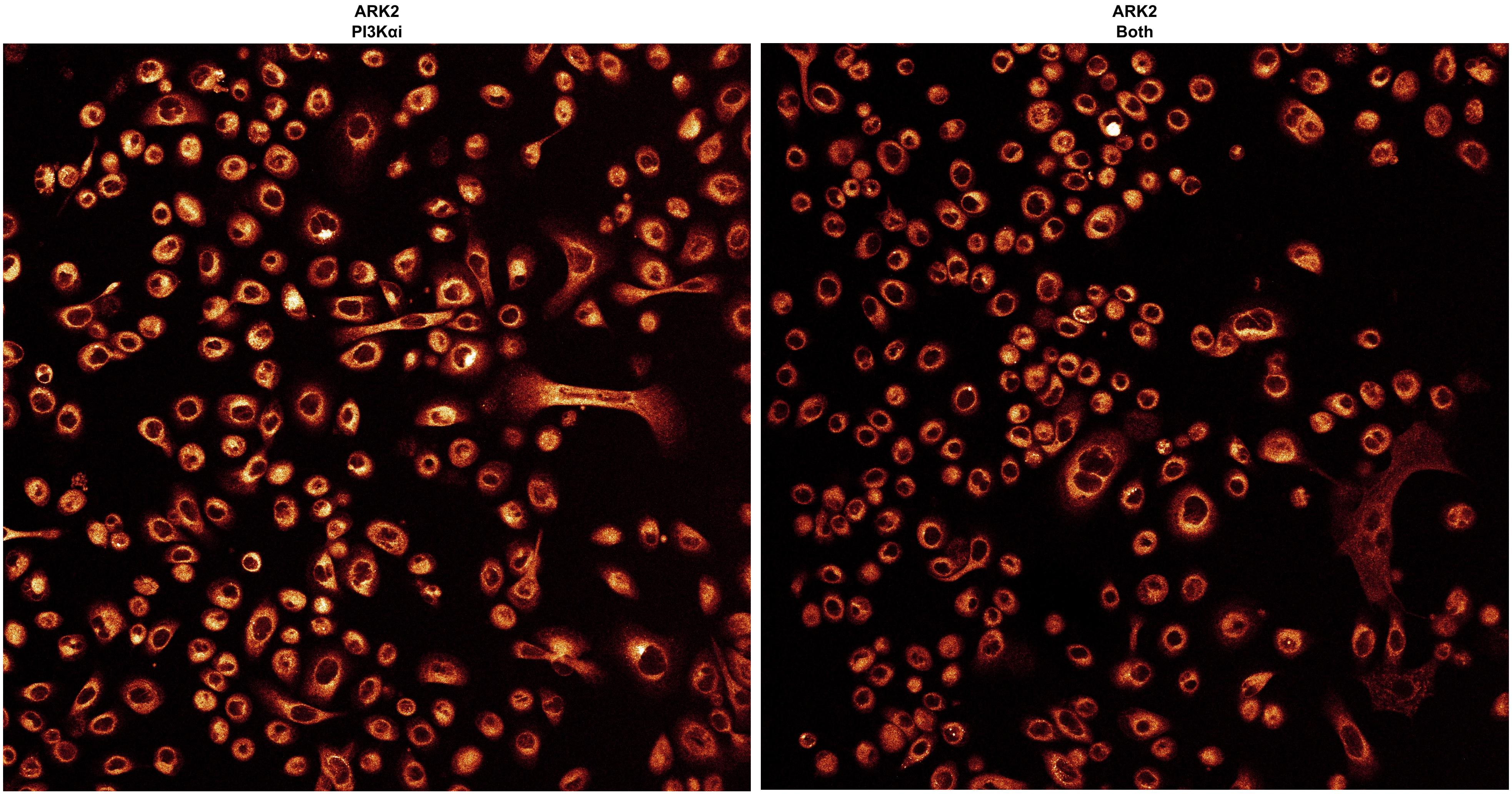

Fig. 4A

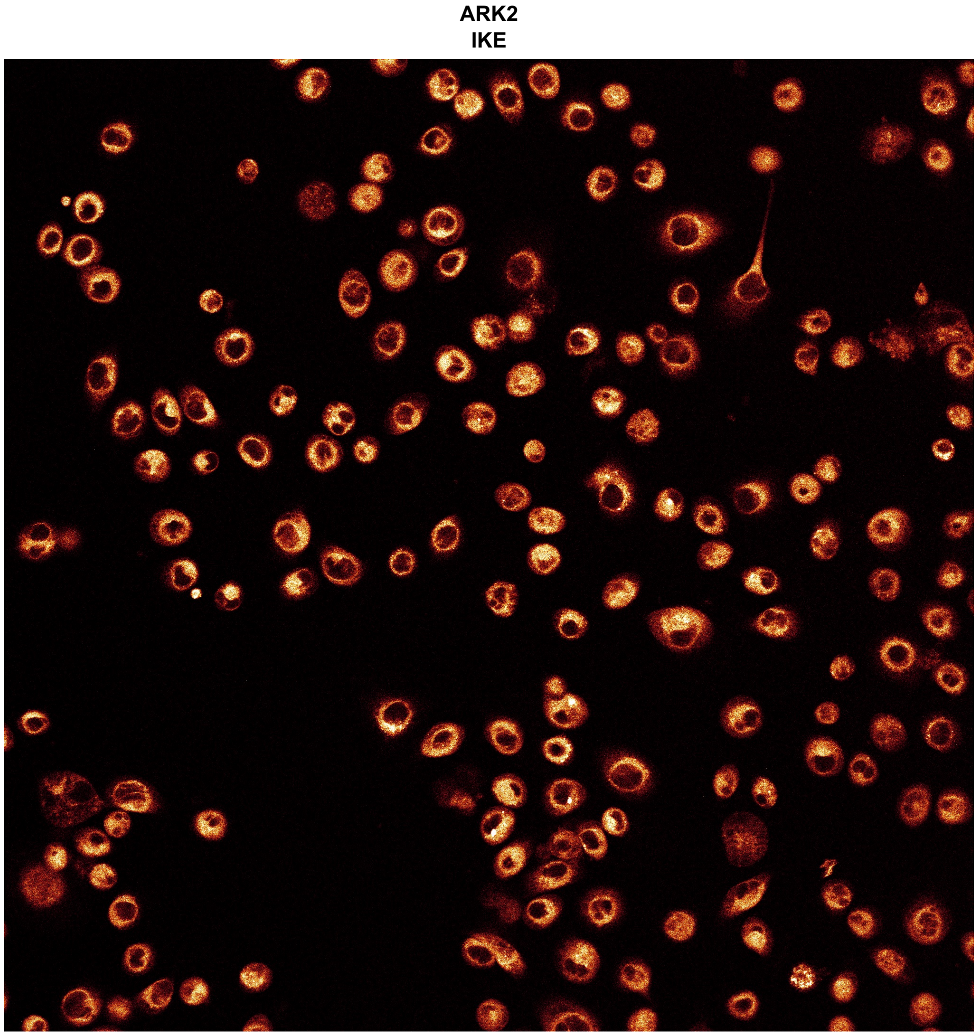

Fig. 4A

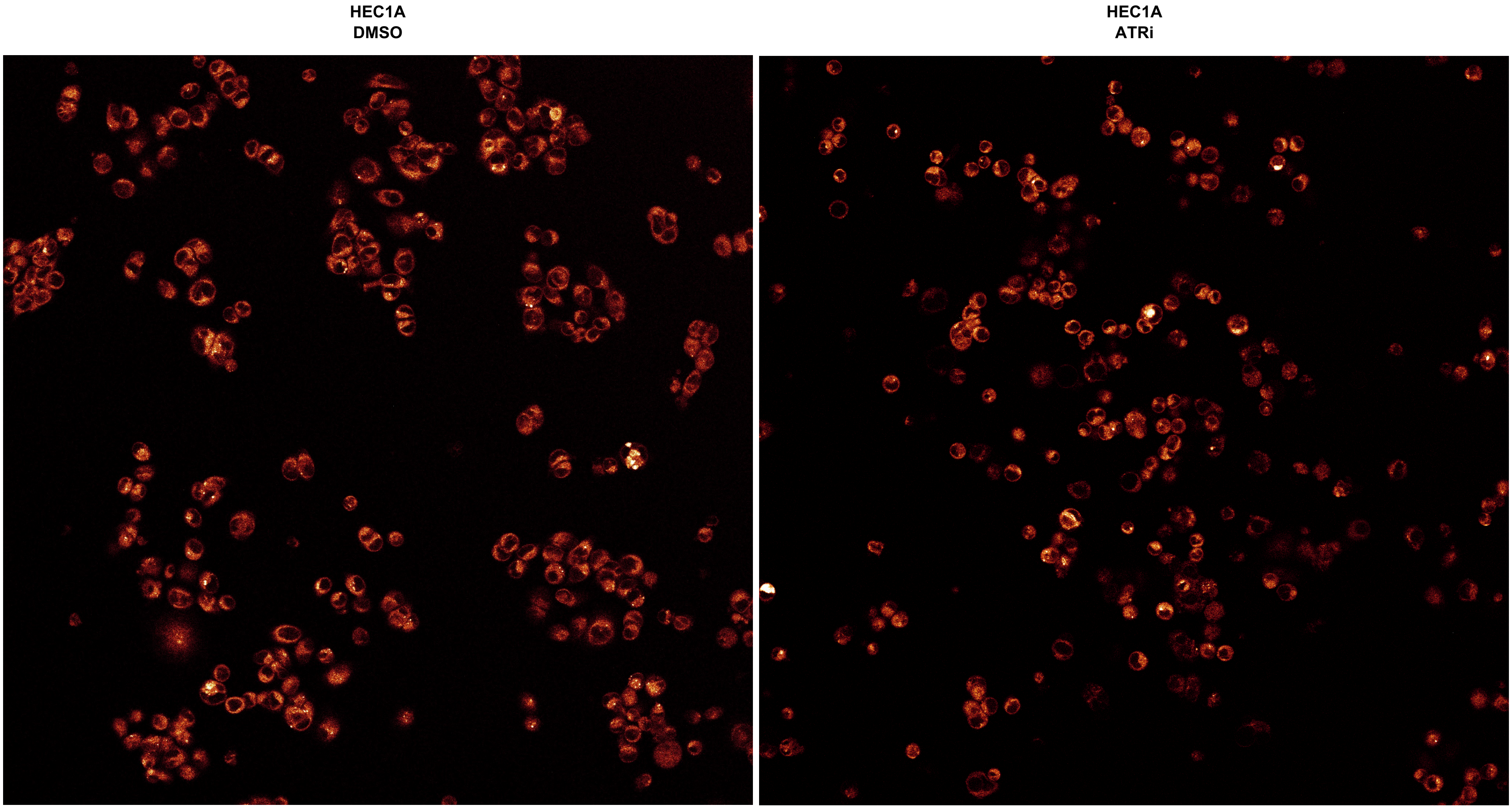

Fig. 4A

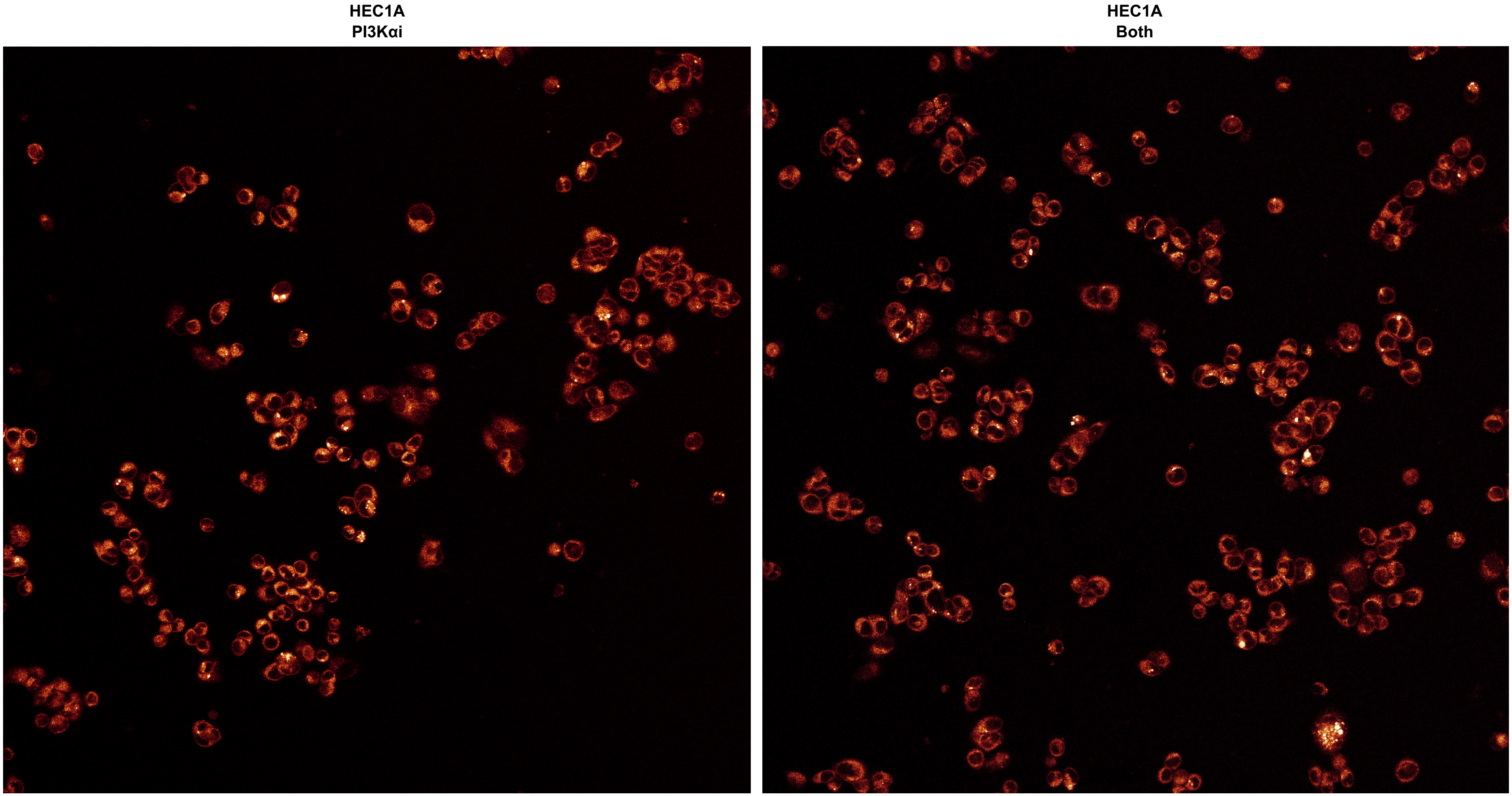

Fig. 4A

HEC1A
IKE

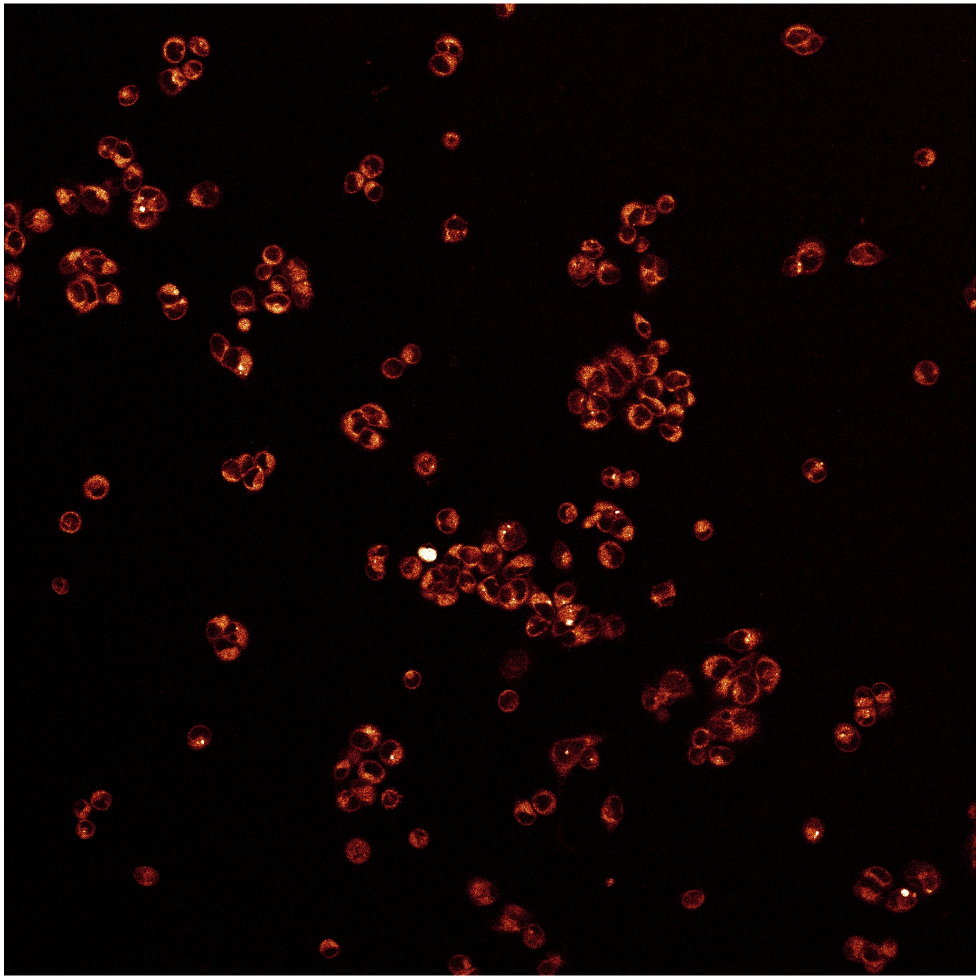

Fig. 4B

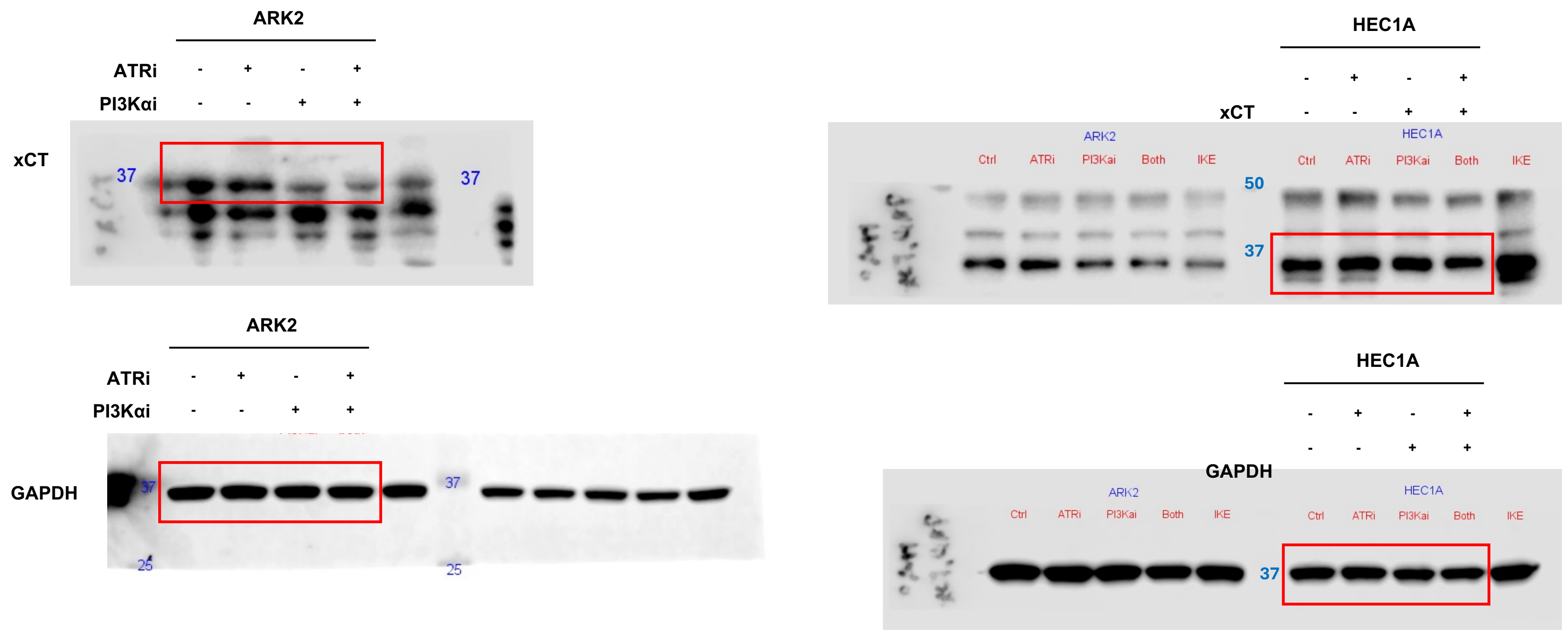
ARK2
ATRi - + - +
PI3Kαi - - + +
xCT
37
37
ARK2
ATRi - + - +
PI3Kαi - - + +
GAPDH
37
25
37
25
HEC1A
- + - +
xCT - - + +
ARK2
HEC1A
Ctrl ATRi PI3Kai Both IKE
Ctrl ATRi PI3Kai Both IKE
50
37
HEC1A
- + - +
- - + +
GAPDH
ARK2
HEC1A
Ctrl ATRi PI3Kai Both IKE
Ctrl ATRi PI3Kai Both IKE
37

Fig. 5B

354T
DMSO

354T
ATRi

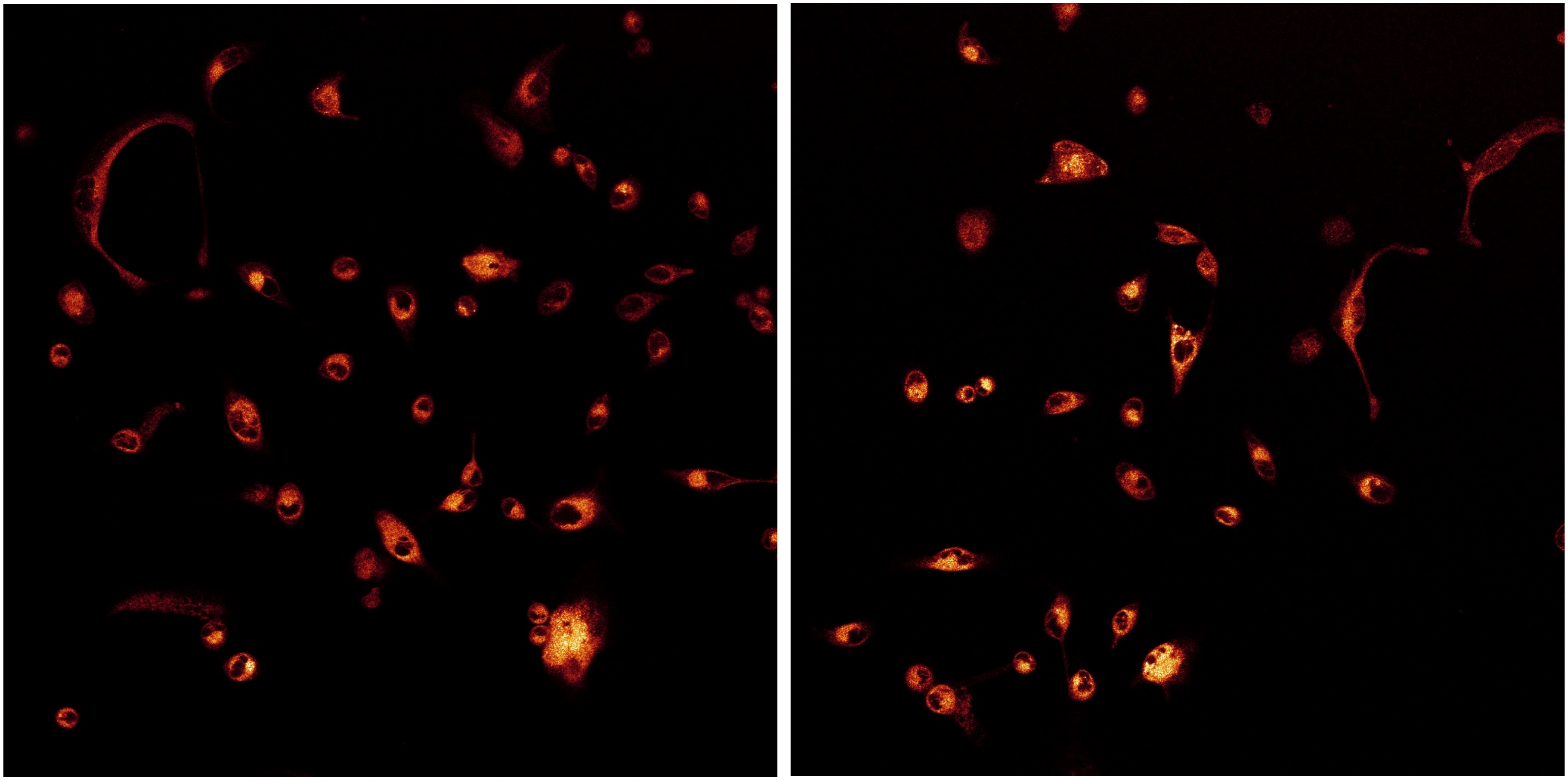

Fig. 5B

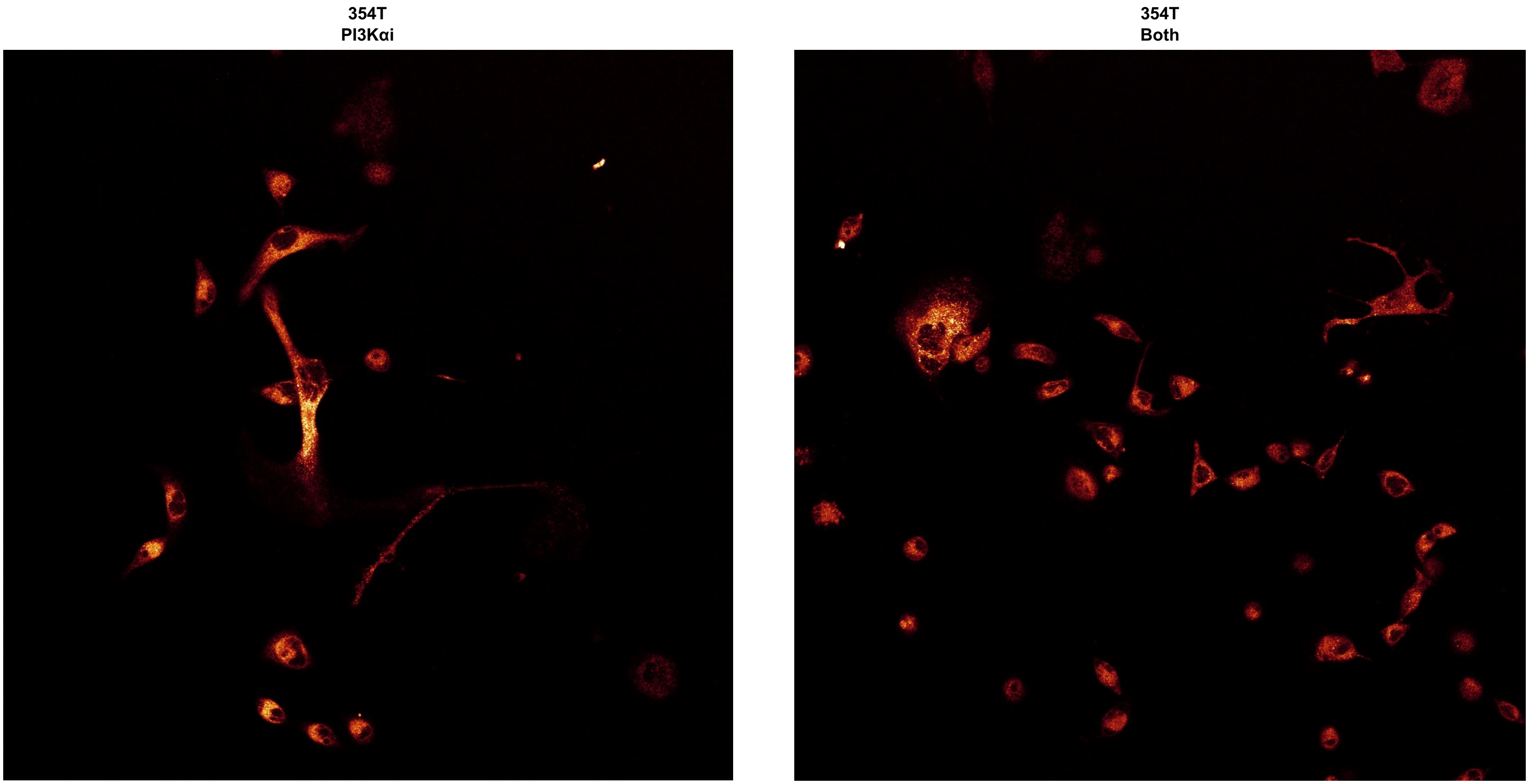

Fig. 5B

354T
IKE

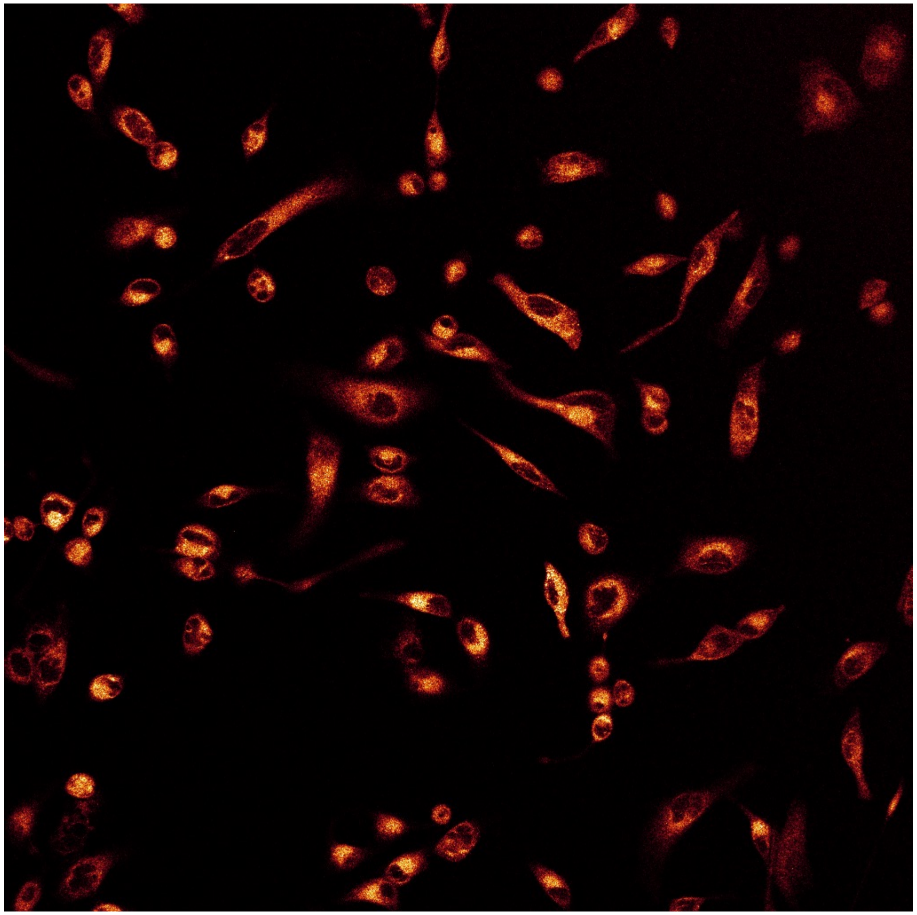

Fig. 5B

144R DMSO | **144R ATRi**

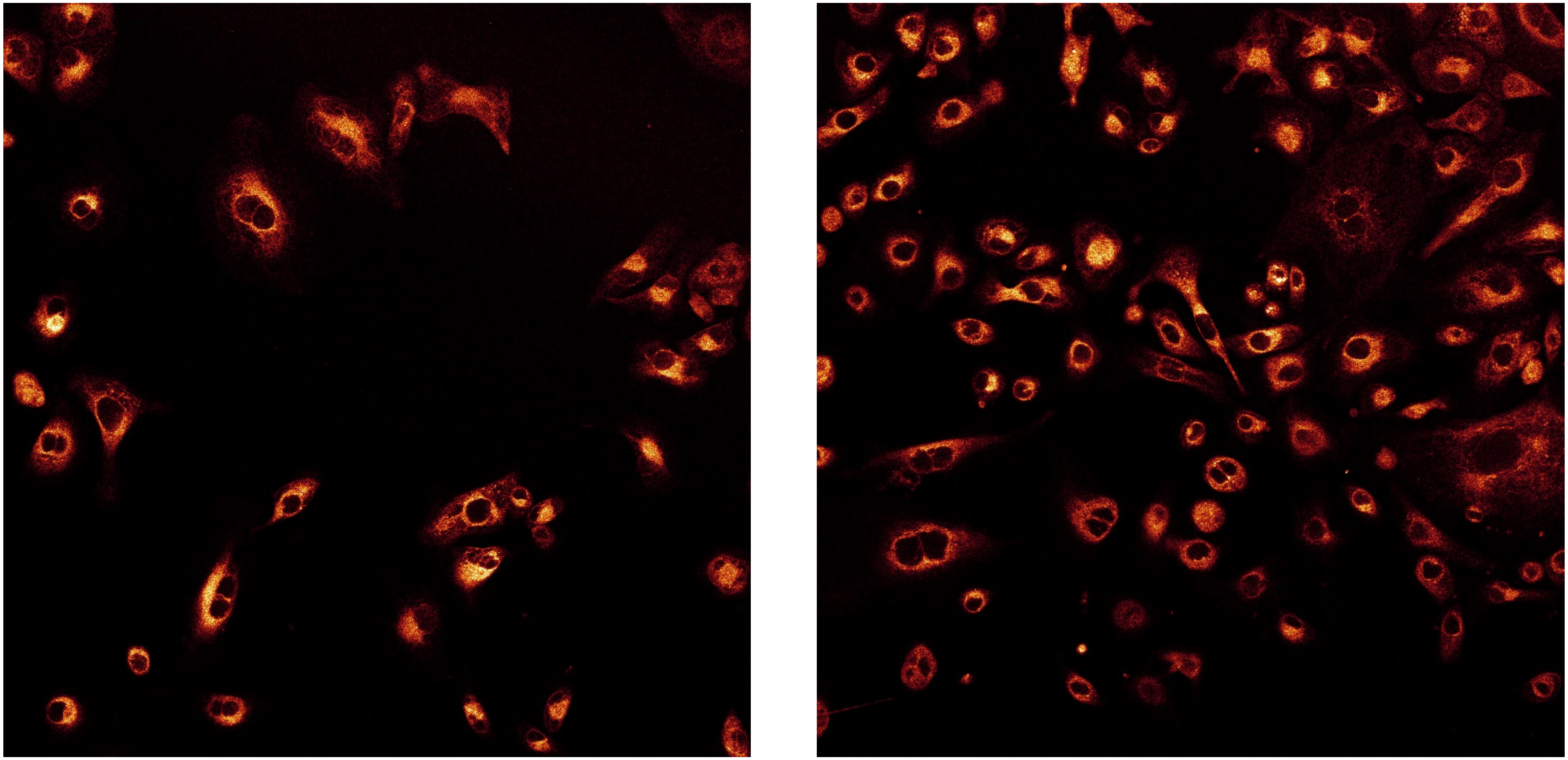

Fig. 5B

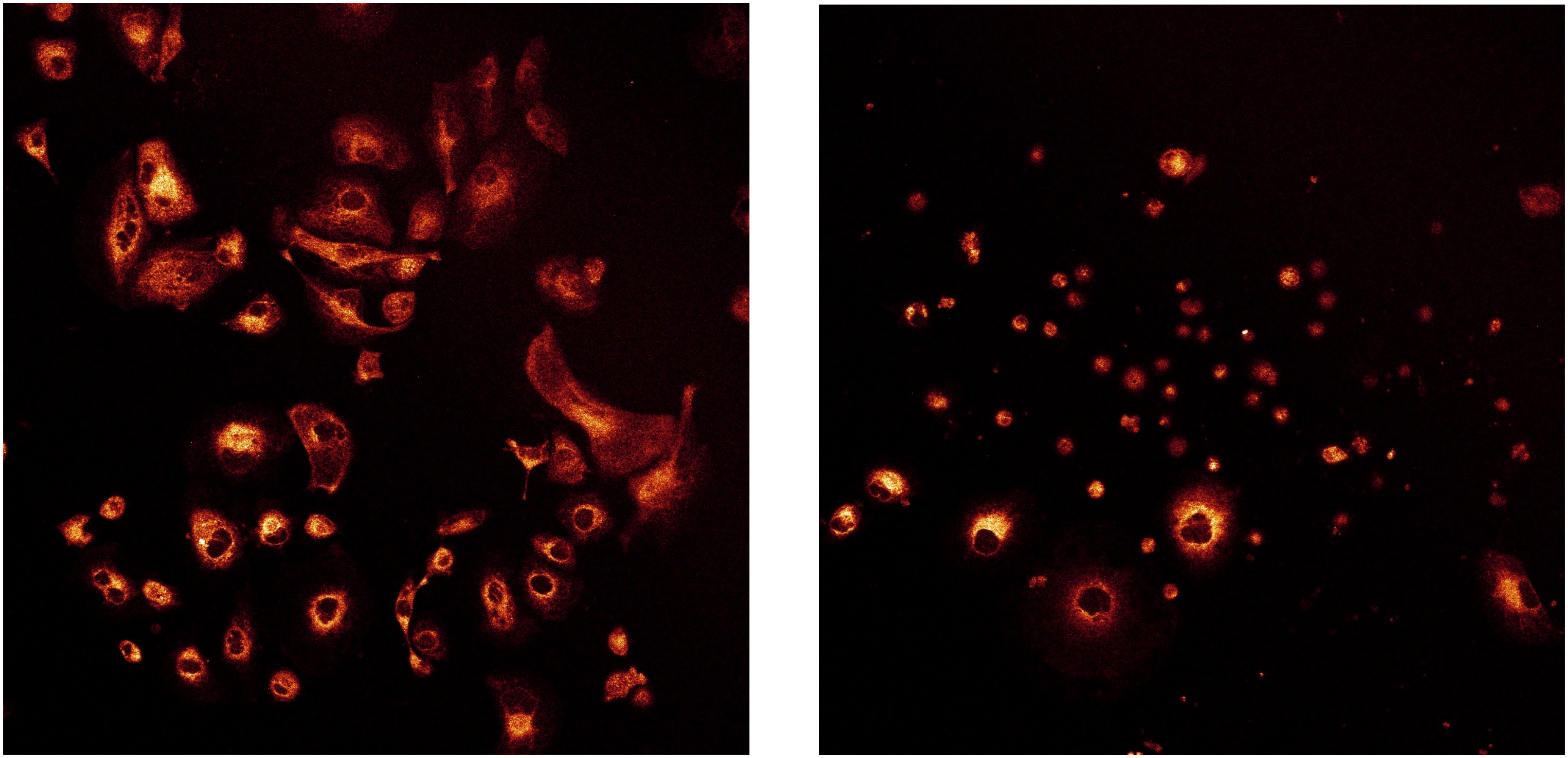

Fig. 5B

144R
IKE

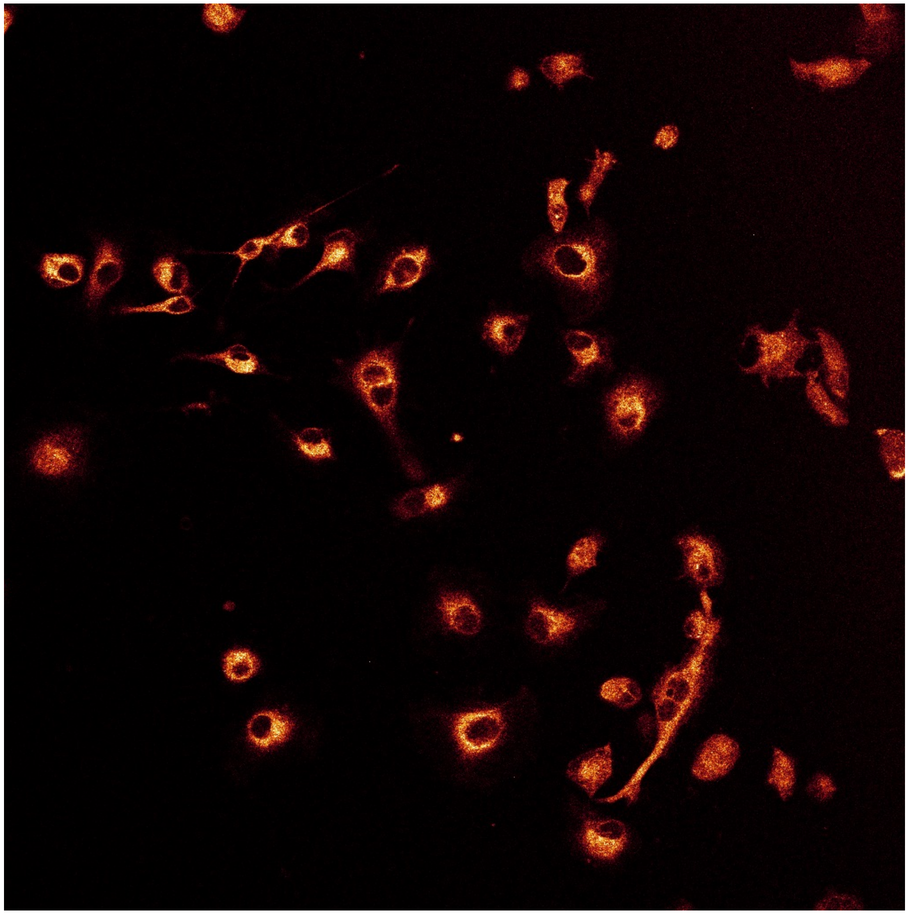

Fig. 5C

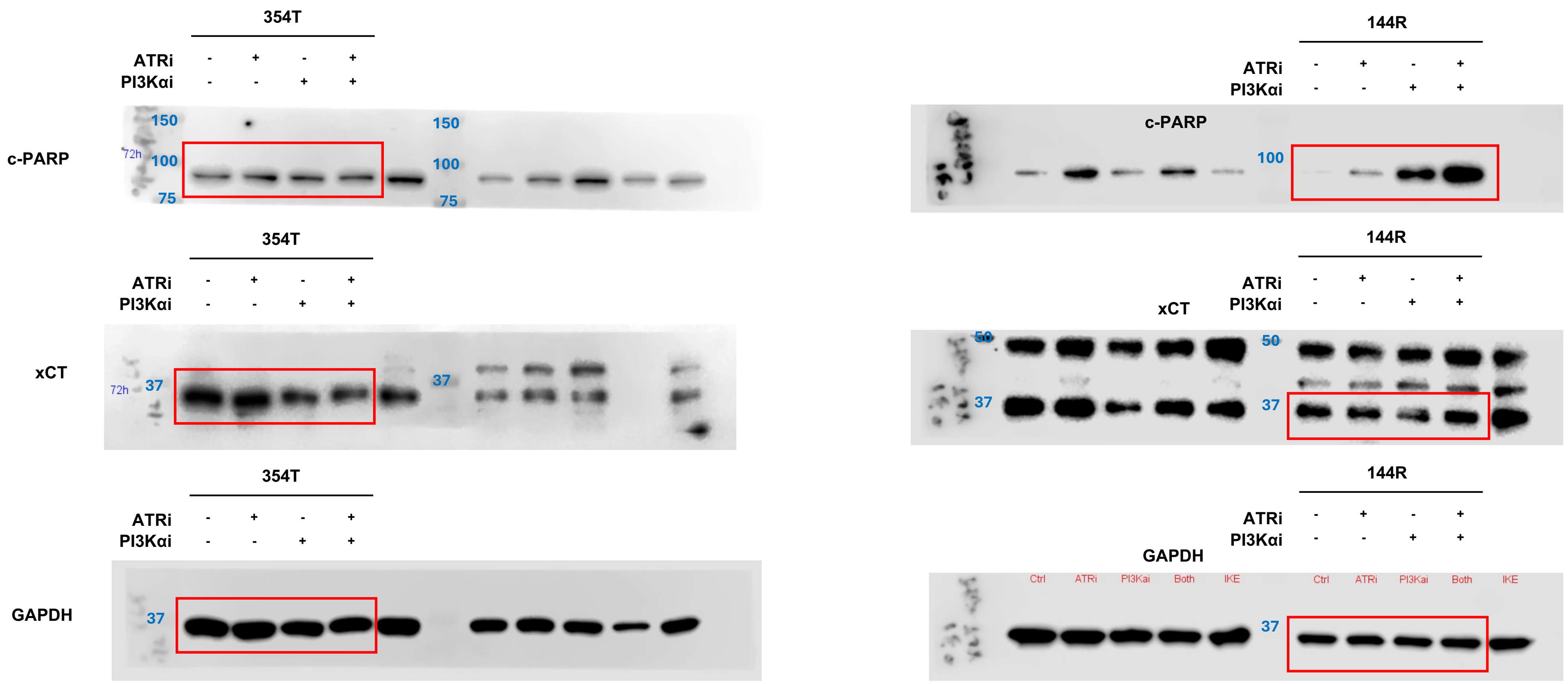
354T
ATRi - + - +
PI3Kαi - - + +
c-PARP
72h
150
100
75
354T
ATRi - + - +
PI3Kαi - - + +
xCT
72h
37
354T
ATRi - + - +
PI3Kαi - - + +
GAPDH
37
144R
ATRi - + - +
PI3Kαi - - + +
c-PARP
100
144R
xCT
ATRi - + - +
PI3Kαi - - + +
50
37
144R
ATRi - + - +
PI3Kαi - - + +
GAPDH
Ctrl
ATRi
PI3Kai
Both
IKE
37

Fig. S2

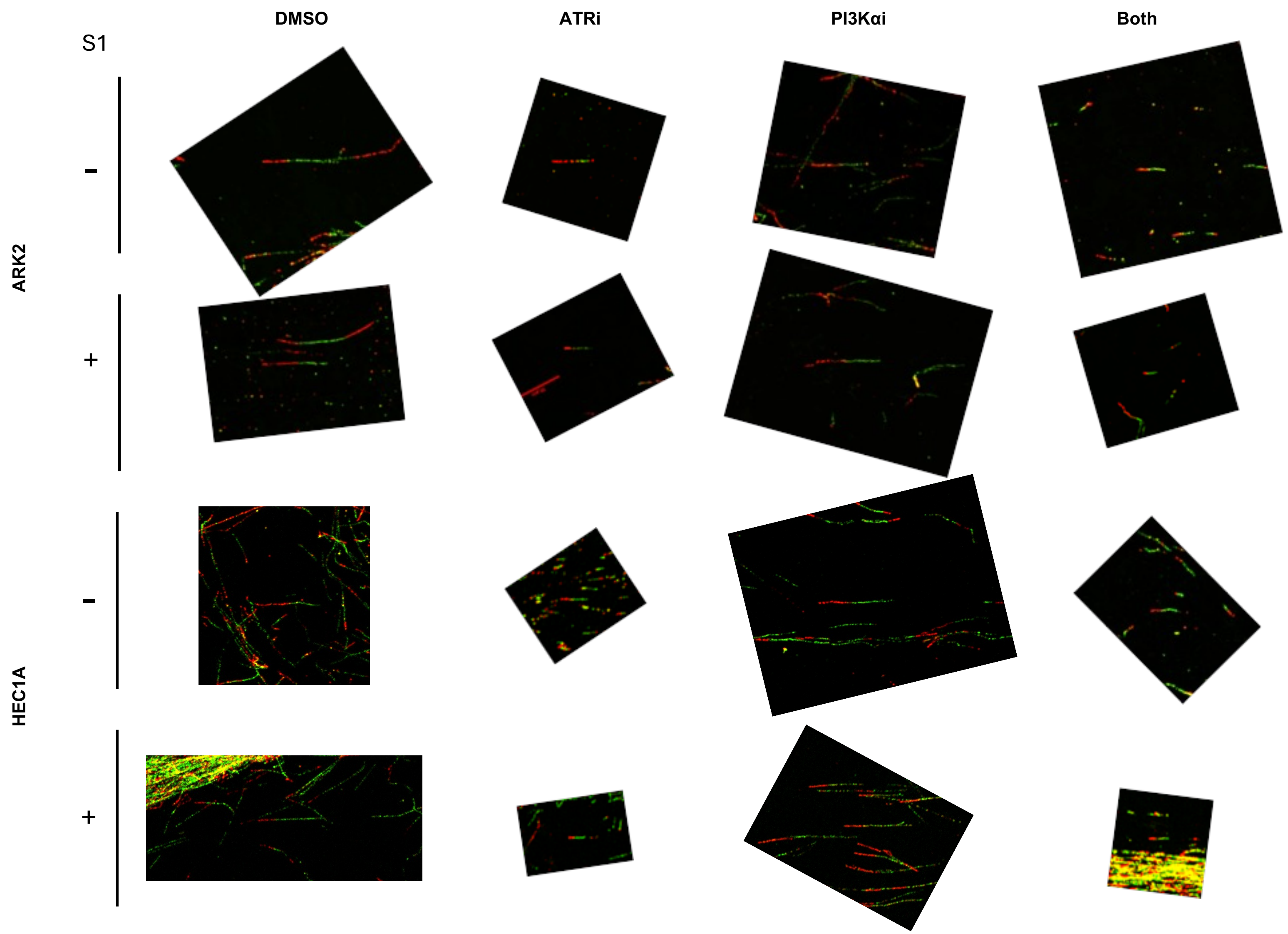

Supplement: Supplementary file 1 [file cancers-18-01064-s001.zip › cancers-4189632-S2.pdf]
